# Nucleotide substitutions in the *mexR*, *nalC* and *nalD* regulator genes of the MexAB-OprM efflux pump are maintained in *Pseudomonas aeruginosa g*enetic lineages

**DOI:** 10.1371/journal.pone.0266742

**Published:** 2022-05-10

**Authors:** Pamela Aguilar-Rodea, Gerardo Zúñiga, René Cerritos, Benjamín Antonio Rodríguez-Espino, Uriel Gomez-Ramirez, Carolina G. Nolasco-Romero, Beatriz López-Marceliano, Gerardo E. Rodea, Sandra Mendoza-Elizalde, Alfonso Reyes-López, Héctor Olivares Clavijo, Juan Carlos Vigueras Galindo, Norma Velázquez-Guadarrama, Irma Rosas-Pérez

**Affiliations:** 1 Posgrado en Ciencias de la Tierra, Centro de Ciencias de la Atmósfera, Universidad Nacional Autónoma de México, Ciudad de México, México; 2 Unidad de Investigación en Enfermedades Infecciosas Área de Genética Bacteriana, Hospital Infantil de México Federico Gómez, Ciudad de México, México; 3 Laboratorio de Aerobiología, Centro de Ciencias de la Atmósfera, Universidad Nacional Autónoma de México, Ciudad de México, México; 4 Laboratorio de Variación Biológica y Evolución, Departamento de Zoología, Escuela Nacional de Ciencias Biológicas, Instituto Politécnico Nacional, Ciudad de México, México; 5 Centro de Investigación en Políticas Población y Salud, Centro de Ciencias de la Complejidad, Universidad Nacional Autónoma de México, Ciudad de México, México; 6 Laboratorio de Investigación y Diagnóstico en Nefrología y Metabolismo Mineral Óseo, Hospital Infantil de México Federico Gómez, Ciudad de México, México; 7 Programa de Posgrado en Ciencias Químicobiologicas, Escuela Nacional de Ciencias Biológicas. Instituto Politécnico Nacional, Ciudad de México, México; 8 Centro de Estudios Económicos y Sociales en Salud, Dirección de Investigación, Hospital Infantil de México Federico Gómez, Ciudad de México, México; 9 Hemerobiblioteca, Hospital Infantil de México Federico Gómez, Ciudad de México, México; University of Newcastle, Australia, AUSTRALIA

## Abstract

*Pseudomonas aeruginosa* has different resistant mechanisms including the constitutive MexAB-OprM efflux pump. Single nucleotide polymorphisms (SNPs) in the *mexR*, *nalC*, and *nalD* repressors of this efflux pump can contribute to antimicrobial resistance; however, it is unknown whether these changes are mainly related to genetic lineages or environmental pressure. This study identifies SNPs in the *mexR*, *nalC*, and *nalD* genes in clinical and environmental isolates of *P*. *aeruginosa* (including high-risk clones). Ninety-one *P*. *aeruginosa* strains were classified according to their resistance to antibiotics, typified by multilocus sequencing, and *mexR*, *nalC*, and *nalD* genes sequenced for SNPs identification. The *mexAB-oprM* transcript expression was determined. The 96.7% of the strains were classified as multidrug resistant. Eight strains produced serine carbapenemases, and 11 strains metallo-β-lactamases. Twenty-three new STs and high-risk clones ST111 and ST233 were identified. SNPs in the *mexR*, *nalC*, and *nalD* genes revealed 27 different haplotypes (patterns). Sixty-two mutational changes were identified, 13 non-synonymous. Haplotype 1 was the most frequent (n = 40), and mainly identified in strains ST1725 (33/40), with 57.5% pan drug resistant strains, 36.5% extensive drug resistant and two strains exhibiting serin-carbapenemases. Haplotype 12 (n = 9) was identified in ST233 and phylogenetically related STs, with 100% of the strains exhibiting XDR and 90% producing metallo-β-lactamases. Haplotype 5 was highly associated with XDR and related to dead when compared to ST1725 and ST233 (RRR 23.34; p = 0.009 and RRR 32.01; p = 0.025). A significant relationship between the *mexR-nalC-nalD* haplotypes and phylogenetically related STs was observed, suggesting mutational changes in these repressors are highly maintained within genetic lineages. In addition, phylogenetically related STs showed similar resistant profiles; however, the resistance was (likely or partly) attributed to the MexAB-OprM efflux pump in 56% of the strains (only 45.05% showed *mexA* overtranscription), in the remaining strains the resistance could be attributed to carbapenemases or mechanisms including other pumps, since same SNPs in the repressor genes gave rise to different resistance profiles.

## Background

*Pseudomonas aeruginosa*, a free-living microorganism, is a major opportunistic pathogen involved in healthcare-associated infections with high mortality rates [1, 2]. Worldwide, *P*. *aeruginosa* is of major public health importance because it exhibits elevated resistance to nearly all types of antibiotics used for its mitigation [3, 4] and the distribution of high-risk clones is increasing [5].

*P*. *aeruginosa* is known to have a non-clonal population structure with some outstanding sequence types (ST). The constant appearance of new variants has allowed for the observation that while this non-clonal structure is maintained in sensitive strains, multi-resistant strains primarily exhibit a clonal structure [5, 6]. Worldwide, ST111, ST146, ST175, ST233, and ST235 are the major high-risk clones associated with multidrug resistance (MDR) and extensive drug resistance (XDR); other clones, including ST357 and ST664 have also been described but with less frequency [5, 7–9].

The multi-antibiotic resistance of clinical *P*. *aeruginosa* strains lies from the combined effects of its intrinsic resistance, attributed to the low permeability of its external membrane and efflux pumps [10], and acquired resistance, principally through mutational changes or genetic material uptake [11]. The MexAB-OprM efflux pump is a major contributor to multi-resistance that confers advantages to *P*. *aeruginosa* by expelling a diverse range of agents: fluoroquinolones, β-lactamics, β-lactamase inhibitors, extended spectrum cephalosporins, carbapenems, tetracycline, macrolides, chloramphenicol, novobiocin, trimethoprim, sulfonamides, dyes, detergents, fatty acids biosynthesis inhibitors, organic solvents, and homoserine lactones associated with cell–cell signaling systems and virulence determinants [12–16], and its overexpression has been observed in strains bearing mutations in its regulatory genes (*mexR*, *nalC*, and *nalD*) [17–19].

The MexAB-OprM efflux pump belongs to the resistance–nodulation–cell division family and is a complex that comprises three components: a cytoplasmic membrane transporter (MexB), a membrane fusion protein (MexA), and an external membrane pore (OprM). This system allows antimicrobials and other toxic compounds transportation from the cytoplasm to the extracellular environment [10, 20].

Multidrug efflux pumps belonging to the RND family are mainly regulated at the transcriptional level, where regulators bind to DNA sequences that are located near or within the promoter regions of the genes they modulate [21]. Generally, transcriptional regulators contain one DNA-binding motif which consists of an α-helix-turn-α-helix motif. The second helix inserts into the major groove of target DNA and determine the binding specificity of the regulator; however, a third helix is needed to stabilize the DNA-binding motif [21]. In some cases, a local transcriptional regulator gene is linked to the same operon that encodes the efflux system it modulates, as occurs with *mexR*. In other cases, regulatory genes are dispersed throughout the chromosome and could regulate multiple genes simultaneously (*nalC*) [22].

Constitutive expression of the MexAB-OprM efflux pump maintains basal levels of the pump and is mediated by the repressor genes *mexR*, *nalC*, and *nalD*. The *mexAB-oprM* operon that encodes the efflux pump is regulated directly by the transcriptional repressors MexR and NalD and indirectly by NalC, which represses ArmR protein expression (MexR anti-repressor) to de-repress efflux pump expression. Mutations in the *mexR*, *nalC*, and *nalD* genes can impair their function, favoring MexAB-OprM efflux pump overexpression with a consequent increase in bacterial resistance [10, 23, 24].

Recent studies suggest SNPs in efflux pumps and porins are responsible for the generation of high-risk clones with MDR phenotypes [5, 25, 26]; however, in the case of the MexAB-OprM efflux pump, only a small number of studies have investigated the relationship between SNPs in the three repressor genes (*mexR*, *nalC*, and *nalD*) and the MDR phenotype [27, 28]. Mutational changes in the MexAB-OprM regulators may be associated with pressure exerted by the environment in which the strains are found, such as antibiotics, reactive oxygen species, detergents, among others [29, 30]; however, it is unknown whether ST´s or high-risk clones of different origins present the same changes in these repressors regardless their genetic lineage.

This study aims to identify SNPs in the *mexR*, *nalC*, and *nalD* repressor genes of the MexAB-OprM efflux pump in clinical and environmental isolates of *P*. *aeruginosa* (including high-risk clones) to discern whether these mutational changes may be associated with the pressure exerted by environmental conditions or are mainly related to genetic lineages.

## Methods

### Bacterial isolates

91 *P*. *aeruginosa* strains were analyzed: 77 strains of nosocomial origin (1H-77H), isolated at the Central Laboratory of the Hospital Infantil de México Federico Gomez during 2007–2015, and responsible for a high mortality rate (18.64%) during 2007–2013 [6], and 14 of environmental origin (1A-14A), isolated by the Aerobiology Laboratory at the Centro de Ciencias de la Atmósfera, UNAM during 2014. The nosocomial isolates were principally recovered from urine (62.34%; n = 48) and blood (19.48%; n = 15). The environmental isolates were mainly obtained from soil (42.86%; n = 6), water (35.71%; n = 5), and plants (21.43%; n = 3). Most strains were obtained from the following hospital wards: nephrology, 19.49% (n = 15); emergency, 15.58% (n = 12); and surgical therapy, 11.69% (n = 9).

Strains were taxonomically identified using the automated system MALDI-TOF (Biomerieux Marcy l’Etoile, France).

Reference strains *Escherichia coli* ATCC25922, *Pseudomonas aeruginosa* ATCC 27853 (American Type Culture Collection, Manassas, VA, USA) and *Klebsiella pneumoniae* NCTC 13438 were used as controls to validate the different methodologies.

### Susceptibility profiles

The susceptibility profiles of 58 *P*. *aeruginosa* strains were reported by Aguilar-Rodea *et al*., 2017 [6]. For the remaining 33 strains, the susceptibility profiles were determined according to the minimal inhibitory concentration (MIC) for nine different antibiotic categories [31] using the agar dilution method described by the Clinical and laboratory Standards Institute (CLSI) (2020) [32]. The antibiotics tested were gentamicin (GEN), tobramycin (TOB), amikacin (AK), imipenem (IMI), meropenem, (MEM), ceftazidime (CAZ), cefepime (CPM), ciprofloxacin (CIP), levofloxacin (LEV), carbenicillin (CB), piperacillin/tazobactam (P/T), aztreonam (AZT), fosfomycin (FOS) and, colistin (CS). All the antibiotics used were pure salts (Sigma-Aldrich, St. Louis, M.O). The reference strains used for validation of the technique included *Pseudomonas aeruginosa* ATCC 27853 and *Escherichia coli* ATCC 25922 (American Type Culture Collection, Manassas, VA, USA). The MIC interpretative criteria was performed according to CLSI (2020) [32]. For FOS: as no values were reported, *E*. *coli* breakpoints were taken into consideration S ≤64, I 128, R ≥256; CS: I ≤2, R ≥4 [33].

The strains were classified as follows: sensitive (S), resistant (R), multidrug resistant (MDR), extensively drug resistant (XDR), and pan drug resistant (PDR), according to the CLSI (2020) breakpoints and the criteria described by Magiorakos *et al*., 2012 [31, 32]. MDR strains were defined as non-susceptible to at least one agent in 3 or more antimicrobial categories; XDR, non-susceptible to at least one agent in all but two or fewer antimicrobial categories; PDR, non-susceptible to all agents in all antimicrobial categories.

### Identification of carbapenemase-producing *P*. *aeruginosa*

Carbapenemase-producing *P*. *aeruginosa* were screened using the phenotypic technique βCARBA Test (BIO-RAD, France). This assessment is based on the color change of a pH indicator following hydrolysis of the β-lactam ring in carbapenem. For this test, all carbapenem-resistant isolates were incubated at 37°C for 24 h on Mueller–Hinton agar plates. Isolated colonies were recovered on a calibrated loop, resuspended in a bacterial protein extraction reagent, and further incubated for 30 min at 37°C. Red color indicates a positive test, while no color change indicates negative. *Escherichia coli* ATCC 25922 was used as negative control, and *Klebsiella pneumoniae* NCTC 13438 (KPC-3 carbapenemase) served as the positive control [34].

In carbapenem-positive strains, serine carbapenemase and metallo-β-lactamase were evaluated using the modified carbapenem inactivation method (mCIM) and the EDTA-modified carbapenem inactivation method (eCIM) as described by the CLSI, 2020 [32].

For the mCIM test, a 10-μL loop of *P*. *aeruginosa* from an overnight blood agar plate was resuspended in 2 mL of Mueller Hinton broth, and a 10-μg meropenem disk was added following incubation for 4 h at 37°C. A suspension of *E*. *coli* ATCC 25922 (0.5 McFarland, meropenem susceptible MIC ≤ 2 μg/mL) was inoculated onto a Mueller–Hinton plate, and the previously-treated meropenem disk was added. For the eCIM test, 2 mL Mueller–Hinton broth with *P*. *aeruginosa* was prepared, and a 10-μg meropenem disk with 20 μL of 0.5 M EDTA was added and incubated for 4 h at 37°C. Both meropenem disks were placed on one Mueller–Hinton plate inoculated with the meropenem susceptible reference strain and incubated for 24 h at 37°C. The inhibition zones were measured and interpreted using the following guidelines established by CLSI, 2020 [32].

### MexAB-OprM efflux pump phenotypic detection

Phenotypic activity of the MexAB-OprM efflux pump was assessed in the 91 *P*. *aeruginosa* strains and confirmed as a 4-fold decrease in MIC value for CB in the presence of Phe-Arg-β-naphthylamine (PaβN) inhibitor relative to that in the absence of the inhibitor. For this technique, the MexB-specific substrate CB was used as the reporter antibiotic [35]. The MIC was determined for each strain in the absence/presence of the efflux inhibitor PaβN (50 μg/mL). At this concentration, the inhibitor completely restored the susceptibility of the control strain *P*. *aeruginosa* ATCC 27853 (American Type Culture Collection, Manassas, VA) to the reporter antibiotic and did not inhibit bacterial growth [36, 37]. All isolates grew in the presence of PaβN (50 μg/mL). The MexAB-OprM efflux pump was considered as the most likely cause of the elevation of the MIC (+) if the MIC value for **CB -PaβN** was at least 2 log_2_ dilutions higher than in the reference strain (PAO1: MIC 64 μg/ml), and the MIC for **CB +PaβN** was lower than that measured in the reference strain (PAO1: MIC 64 μg/ml) or α 1 dilution; if the MIC values for **CB +PaβN** remained elevated compared with the reference strain, the MexAB-OprM efflux pump was contributing in the elevation of the MIC (*); and if there was a difference of 1 dilution between **CB -PaβN** and **CB +PaβN** or no difference, the MexAB-OprM efflux pump was not the cause of the elevation of the MIC (-) [35].

### MexAB-OprM efflux pump genotypic detection

*P*. *aeruginosa* from an overnight blood agar plate was resuspended in 2 mL of Mueller Hinton broth (0.5 McFarland) and incubated at 37°C for 16-24h (synchronization phase) (each strain in duplicate). A 1:100 dilution was made, and bacteria was harvested at the late log-phase of growth. Total RNA was isolated using TRIzol Reagent (Zymo Research, USA) followed by DNase I treatment (Thermo Scientific, USA). Synthesis of cDNA was performed using the ZymoScript RT PreMix Kit (Zymo Research, USA) following the manufacturer´s protocol.

The following primers: *mexA* (116 bp): F´5´-GTG AAC GCG CAG AAC AAG-3´, R´5´-AGG CCT TCG GTA ATG ATC TTG-3´ and *rpsL* (117 bp): F´5´-AAC TCG GCA CTG CGT AAG-3´, R´5´-GCC ACG GAT CAG CAC TAC-3´ were designed using the Primer3 program [38], http://bioinfo.ut.ee/primer3-0.4.0/. For the *mexA* detection, the qPCR SybrMaster highROX Kit (Jenna Bioscience, Germany) was used, and the reaction was carried out in an AriaMx Real-time PCR System (Agilent Technologies, USA) with the following conditions: initial denaturation at 95°C for 1 min, followed by 40 cycles: 95°C denaturation 15 sec, alignment 64.4°C for 20 sec, 72°C extension 10 sec, and a final cycle of 95°C, 64.4°C and 95°C for 30 sec each; with a melting curve between 65-95°C showing continuous fluorescence readings. Real-time PCR determinations were performed in duplicate, a total of 4 data sets for each strain were obtained. For each run, a positive and a negative control were incorporated. *mexAB-oprM* e overtranscription (+) was considered if a >3-fold *mexA* value was obtained compared to the reference strain (PAO1: 1) [35].

The efficiency of the reaction was calculated using the formula E = 10 ^**(−1/slope)**^ for which a standard curve was made with serial dilutions of cDNA (1:2) of the reference strain (PAO1) (in triplicate), the Cq values observed were plotted against the logarithm of the concentration to obtain the slope of each gene (*mexA* and *rpsL*). Gene transcript was normalized to the *rps*L housekeeping gene and transcript levels were calculated as transcript expression ratio compared to the reference strain PAO1; the equation for relative quantification corrected for efficiency (Fold effect = (EmexA^Δ*C*q,mexA(control−sample)^)/ ErpsL^Δ*C*q,rpsL(control−sample)^) was applied [27, 39].

### Bacterial DNA isolation and quantification

*P*. *aeruginosa* was cultured in Mueller Hinton broth at 37°C for 18–20h. Chromosomal DNA was isolated using the Wizard Genomic DNA purification kit (Promega, USA), following the manufacturer´s protocol. DNA quality, integrity, and concentration were confirmed by agarose (1%) gel electrophoresis. DNA concentration and purity were evaluated using an EPOCH spectrophotometer (Biotek, Vermont, USA). High quality DNA was stored at 4°C until used.

### Genotyping via Multilocus Sequence Typing (MLST)

The ST of 58 of the *P*. *aeruginosa* strains was previously determined [6]; for the remaining 33 strains, the same genotyping procedure via MLST was performed. Nested PCR for the metabolic genes *acsA*, *aroE*, *guaA*, *mutL*, *nuoD*, *ppsA*, and *trpE* was carried out using the primers described by Curran *et al*., 2004 [40]. Sequencing of the PCR products was performed in both senses. The obtained sequences were edited and aligned as previously described. The ST of each strain was obtained by BLAST analysis (nucleotide) of each gene compared with the *P*. *aeruginosa* MLST database [41], http://pubmlst.org/paeruginosa/. The new STs were deposited in the *P*. *aeruginosa* MLST data base. Variability parameters were determined as previously described.

### Identification of SNPs in the MexAB-OprM efflux pump repressor genes *mexR*, *nalC*, and *nalD*

The *mexR*, *nalC*, and *nalD* repressor genes were amplified by PCR in a Thermo Hybaid Thermal cycler (PCR Express, California). The following primers *mexR* (597 bp): F´5´-CAGTAAGCGGATACCTGAAAC-3´, R´5´-GGTTGATAAGGTCAACTAAAATAAGC-3´; *nalC* (998 bp): F´5´-GAAACGCTCTCAGCAAACC-3´, R´5´-CACCGAGATCCACCTCAC-3´; and *nalD* (1,035 bp): F´5´-GCATTAGACAAAGGTGGTGTCG-3´, R´5´-GGCAATACCATGCAAGTTTTCAA-3´ were designed using the Primer3 program [38], http://bioinfo.ut.ee/primer3-0.4.0/. All genes were amplified under the following conditions: 96°C initial denaturation for 1 min, followed by 30 cycles: 96°C denaturation 1 min, alignment (*mexR*: 57°C, *nalC*: 57.7°C and *nalD*: 57°C) for 1 min, 72°C extension 1 min and a 72°C final extension for 5 min. PCR products were purified using the ExoSap IT enzyme (Affymetrix, Cleveland OH, USA) according to the manufacturer’s instructions. PCR products were preserved at −20°C.

PCR products were sequenced in both senses using the primers for each gene described above using a Genetic Analyzer 310 sequencer (Applied Biosystems, Foster City, California, USA). Sequence analysis was performed using ClustalW ver. 2.0 [42], http://www.clustal.org/clustal2/, Seaview ver. 4 [43], http://pbil.univ-lyon1.fr/software/seaview.html and FinchTV ver. 1.4.0 [44], http://www.softpedia.com/get/Science-CAD/FinchTV.shtml. *P*. *aeruginosa* PAO1 was used as reference. Nucleotide sequences were translated into amino acid sequences to determine the variability parameters (ratio of non-synonymous to synonymous substitutions, sites of mutational changes, and polymorphisms) using DnaSP ver 5.10.01 [45], http://www.softpedia.com/get/Science-CAD/DnaSP.shtml.

### Phylogenetic analysis

The phylogenetic relationship and evolutionary relationship of the 91 nosocomial and environmental *P*. *aeruginosa* strains were evaluated by the construction of a phylogenetic network using maximum likelihood. To visualize the genomic relationships, a minimum-spanning tree was built from the MLST (ST) sequences using the GrapeTree [46], the phylogenetic inference was performed in PhyloViz Online [47], https://online.phyloviz.net/index softwares. In addition, groups of related STs (clonal complexes) were identified using the BURST analysis. A group of related STs was defined as a profile match at n-3 loci to any other member of the group (n = number of loci in the scheme, MLST = 7); default settings were used to achieve the most stringent definition. The GrapeTree, PhyloViz Online and BURST analysis softwares are available at the *Pseudomonas aeruginosa* PubMLST database [41], http://pubmlst.org/paeruginosa/.

Furthermore, to determine the evolutionary relationships and events of recombination between the STs a phylogenetic network was built from the MLST (ST) of the *P*. *aeruginosa* strains using the neighbor-net algorithm (distance-based-method) implemented in SplitsTree ver.4.0. [48]. The robustness of the network was calculated with a bootstrap test after 1000 pseudo replicates and the inference of recombination events during the generation of allelic variation was estimated with the pairwise homoplasy index test (PHI).

### Genetic diversity

For each of the MLST and MexAB-OprM efflux pump repressor genes, the number and frequency of haplotypes was determined, as well as the estimated nucleotide diversity, including the nucleotide diversity per site (Pi) and expected variation per site assuming a neutral evolution (Eta). The number of substitutions (S) for each gene is reported as well. All data were obtained using DnaSP 5.10.01 [45]. The DnaSP program allows the analysis of DNA polymorphisms using data from several loci by estimating several measures of DNA sequence variation within and between populations.

### Statistical analysis

The nature of the data determined the type of statistical analysis used. Qualitative variables defined subgroups of the total cohort; therefore, associations between variables required the construction of contingency tables to identify association patterns from the counts within these values. Statistical significance was considered as *p* ≤0.05 as determined by Fisher´s exact test using STATA/MP 14.1 [49]. To investigate the effects of explanatory variables on a binary response variable we used logistic regression models, while for a categorical dependent variable with outcomes that have no natural ordering, multinomial logit models were used. All procedures were done with the STATA/MP 14.1 program [49].

For principal component analysis (PCA), a graphic was constructed using RStudio software [50], (http://www.rstudio.com/) to evaluate the relationships between variables (outcome, resistance, ST, MexAB-OprM haplotype, site, year, and ward). Through: (1) determining the covariance matrix of the normalized data, (2) finding the characteristic root and the characteristic vector, (3) determining the contribution rate of the variance of the principal components, (4) removing the main components, and (5) obtaining the principal component value and the integral score.

Statistical significance between *mexA* transcript expression and phenotypic detection of the MexAB-OprM efflux pump, susceptibility profile, and haplotype was evaluated by Kruskal-Wallis equality of populations rank test using the STATA/MP 14.1 program [49].

## Results

### *P*. *aeruginosa* nosocomial strains exhibit more multidrug resistance than environmental strains

The susceptibility profiles of the studied strains to 14 antibiotics in nine categories is shown in Table 1. The 91 strains were classified as S, R, MDR, XDR, or PDR according to their susceptibility profile (Table 1) as determined by the CLSI 2020 standard values and criteria established by Magiorakos *et al*. [27, 28]. 96.7% of the strains (88/91) were classified as multidrug resistant. Of the nosocomial strains, 49.35% (38/77) were classified as XDR; ten strains showed intermediate resistance to P/T, eight strains were AZT sensitive, and six strains were CS intermediate. In addition, 33.77% of the nosocomial strains were classified as PDR (26/77), 15.58% as MDR (12/77), and 1.30% (1/77) as sensitive. Of the environmental strains, 78.57% were classified as MDR (11/14), 7.14% as XDR (1/14), and 14.29% as sensitive (2/14). *P*. *aeruginosa* nosocomial strains were highly associated with the XDR profile compared to the environmental strains and taking the MDR strains as reference (RRR = 34.82; p = 0.001).

**Table 1:** Classification of the *P*. *aeruginosa* strains (origin, haplotype, susceptibility, MexAB-OprM phenotype, *mexA* transcript expression, and carbapenemase production).

			Antibiotics														SP	MexAB-OprM			Transcript expression		Carbapenemase	
ID	H	ST	GEN	TOB	AK	IMI	MEM	CAZ	CPM	P/T	AZT	CIP	LEV	CB*	FOS*	CS		CB—PaβN	CB + PaβN	^*a*^	*mexA*	^*b*^	S	M
18H	**1**	1725	S	S	S	S	S	S	S	I	**R**	I	S	**R**	**R**	S	MDR	*2048*	*256*	*	0.05	-	/	/
54H	**1**	1725	**R**	**R**	**R**	**R**	**R**	**R**	**R**	**R**	**R**	**R**	**R**	**R**	**R**	S	XDR	*2048*	*1024*	-	79.59	+	-	-
3H **†**	**1**	1725	**R**	**R**	**R**	**R**	**R**	**R**	**R**	I	**R**	**R**	**R**	**R**	**R**	**R**	XDR	*2048*	*1024*	-	0.08	-	-	-
14H **†**	**1**	1725	**R**	**R**	I	**R**	**R**	**R**	**R**	I	**R**	**R**	**R**	**R**	**R**	S	XDR	*2048*	*1024*	-	0.02	-	-	-
15H **†**	**1**	1725	**R**	**R**	**R**	**R**	**R**	**R**	**R**	I	**R**	**R**	**R**	**R**	**R**	S	XDR	*2048*	*512*	*	0.03	-	-	-
39H	**1**	1725	**R**	**R**	**R**	S	**R**	I	**R**	**R**	**R**	**R**	S	**R**	**R**	**R**	XDR	*2048*	*512*	*	0.64	-	-	-
55H	**1**	1725	**R**	**R**	**R**	**R**	**R**	**R**	**R**	I	**R**	**R**	**R**	**R**	**R**	**R**	XDR	*2048*	*512*	*	4.31	+	-	-
8H	**1**	1725	**R**	**R**	**R**	**R**	**R**	**R**	**R**	I	**R**	**R**	**R**	**R**	**R**	**R**	XDR	**1024**	**64**	**+**	37.31	+	ND	ND
53H	**1**	1725	**R**	**R**	**R**	**R**	**R**	**R**	**R**	**R**	**R**	**R**	**R**	**R**	**R**	S	XDR	64	4	-	85.93	+	-	-
38H	**1**	1725	**R**	**R**	**R**	**R**	**R**	**R**	**R**	**R**	**R**	**R**	**R**	**R**	**R**	S	XDR	**2048**	**16**	**+**	1.08	-	-	-
43H	**1**	1725	**R**	**R**	**R**	**R**	**R**	**R**	**R**	**R**	**R**	**R**	**R**	**R**	**R**	S	XDR	**2048**	**16**	**+**	0.13	-	-	-
5H	**1**	1725	**R**	**R**	**R**	**R**	**R**	**R**	**R**	I	**R**	**R**	**R**	**R**	**R**	**R**	XDR	**2048**	**8**	**+**	405.31	+	ND	ND
25H	**1**	1725	**R**	**R**	**R**	**R**	**R**	**R**	**R**	**R**	**R**	**R**	I	**R**	**R**	**R**	XDR	**2048**	**8**	**+**	184.10	+	-	-
6H	**1**	1725	**R**	**R**	**R**	**R**	**R**	**R**	**R**	**R**	**R**	**R**	**R**	**R**	**R**	**R**	**PDR**	*2048*	*1024*	-	0.17	-	-	-
7H	**1**	1725	**R**	**R**	**R**	**R**	**R**	**R**	**R**	**R**	**R**	**R**	**R**	**R**	**R**	**R**	**PDR**	*2048*	*1024*	-	0.07	-	-	-
30H	**1**	1725	**R**	**R**	**R**	**R**	**R**	**R**	**R**	**R**	**R**	**R**	**R**	**R**	**R**	**R**	**PDR**	*2048*	*1024*	-	1.66	-	-	-
31H	**1**	1725	**R**	**R**	**R**	**R**	**R**	**R**	**R**	**R**	**R**	**R**	**R**	**R**	**R**	**R**	**PDR**	*2048*	*1024*	-	3.02	+	-	-
37H	**1**	1725	**R**	**R**	**R**	**R**	**R**	**R**	**R**	**R**	**R**	**R**	**R**	**R**	**R**	**R**	**PDR**	*1024*	*512*	-	139.32	+	-	-
11H	**1**	1725	**R**	**R**	**R**	**R**	**R**	**R**	**R**	**R**	**R**	**R**	**R**	**R**	**R**	**R**	**PDR**	*2048*	*512*	*	0.03	-	-	-
12H	**1**	1725	**R**	**R**	**R**	**R**	**R**	**R**	**R**	**R**	**R**	**R**	**R**	**R**	**R**	**R**	**PDR**	*2048*	*512*	*	0.53	-	-	-
26H	**1**	1725	**R**	**R**	**R**	**R**	**R**	**R**	**R**	**R**	**R**	**R**	**R**	**R**	**R**	**R**	**PDR**	*2048*	*512*	*	132.43	+	-	-
36H	**1**	1725	**R**	**R**	**R**	**R**	**R**	**R**	**R**	**R**	**R**	**R**	**R**	**R**	**R**	**R**	**PDR**	*1024*	*256*	*	513.65	+	-	-
40H	**1**	1725	**R**	**R**	**R**	**R**	**R**	**R**	**R**	**R**	**R**	**R**	**R**	**R**	**R**	**R**	**PDR**	*2048*	*512*	*	0.10	-	-	-
41H	**1**	1725	**R**	**R**	**R**	**R**	**R**	**R**	**R**	**R**	**R**	**R**	**R**	**R**	**R**	**R**	**PDR**	**1024**	**16**	**+**	3.82	+	-	-
50H	**1**	1725	**R**	**R**	**R**	**R**	**R**	**R**	**R**	**R**	**R**	**R**	**R**	**R**	**R**	**R**	**PDR**	**2048**	**32**	**+**	1.84	-	-	-
33H	**1**	1725	**R**	**R**	**R**	**R**	**R**	**R**	**R**	**R**	**R**	**R**	**R**	**R**	**R**	**R**	**PDR**	**2048**	**16**	**+**	0.05	-	-	-
48H	**1**	1725	**R**	**R**	**R**	**R**	**R**	**R**	**R**	**R**	**R**	**R**	**R**	**R**	**R**	**R**	**PDR**	**2048**	**16**	**+**	1.97	-	+	-
4H	**1**	1725	**R**	**R**	**R**	**R**	**R**	**R**	**R**	**R**	**R**	**R**	**R**	**R**	**R**	**R**	**PDR**	**2048**	**8**	**+**	0.07	-	ND	ND
16H	**1**	1725	**R**	**R**	**R**	**R**	**R**	**R**	**R**	**R**	**R**	**R**	**R**	**R**	**R**	**R**	**PDR**	**2048**	**8**	**+**	7.06	+	-	-
42H	**1**	1725	**R**	**R**	**R**	**R**	**R**	**R**	**R**	**R**	**R**	**R**	**R**	**R**	**R**	**R**	**PDR**	**2048**	**8**	**+**	0.18	-	-	-
49H	**1**	1725	**R**	**R**	**R**	**R**	**R**	**R**	**R**	**R**	**R**	**R**	**R**	**R**	**R**	**R**	**PDR**	**1024**	**4**	**+**	0.15	-	ND	ND
35H **†**	**1**	1725	**R**	**R**	**R**	**R**	**R**	**R**	**R**	**R**	**R**	**R**	**R**	**R**	**R**	**R**	**PDR**	**2048**	**4**	**+**	0.35	-	-	-
51H	**1**	1725	**R**	**R**	**R**	**R**	**R**	**R**	**R**	**R**	**R**	**R**	**R**	**R**	**R**	**R**	**PDR**	**2048**	**4**	**+**	0.26	-	+	-
46H	**1**	2244	**R**	**R**	S	**R**	**R**	**R**	**R**	**R**	**R**	**R**	**R**	**R**	**R**	**R**	XDR	1024	1024	-	0.82	-	+	-
47H †	**1**	2245	**R**	**R**	**R**	**R**	**R**	**R**	**R**	I	**R**	**R**	**R**	**R**	**R**	**R**	XDR	**2048**	**32**	**+**	6.40	+	-	-
52H	**1**	2247	**R**	**R**	**R**	**R**	**R**	**R**	**R**	**R**	**R**	**R**	**R**	**R**	**R**	S	XDR	**1024**	**4**	**+**	1.00	-	ND	ND
45H	**1**	2243	**R**	**R**	**R**	**R**	**R**	**R**	**R**	**R**	**R**	**R**	**R**	**R**	**R**	**R**	**PDR**	**512**	**32**	**+**	14.07	+	ND	ND
1H	**1**	1723	**R**	**R**	**R**	**R**	**R**	**R**	**R**	**R**	**R**	**R**	**R**	**R**	**R**	**R**	**PDR**	**2048**	**32**	**+**	0.04	-	-	-
32H	**1**	1730	**R**	**R**	**R**	**R**	**R**	**R**	**R**	**R**	**R**	**R**	**R**	**R**	**R**	**R**	**PDR**	**2048**	**4**	**+**	33.00	+	ND	ND
12A	**1**	111	S	S	S	S	I	S	S	S	S	I	S	**R**	**R**	**R**	MDR	512	512	-	0.07	-	/	/
56H	2	1725	**R**	**R**	**R**	**R**	**R**	**R**	**R**	**R**	**R**	**R**	**R**	**R**	**R**	**R**	**PDR**	**512**	**4**	**+**	4.66	+	-	-
44H	3	2246	**R**	**R**	**R**	**R**	**R**	**R**	**R**	I	**R**	**R**	**R**	**R**	**R**	**R**	XDR	*2048*	*512*	*	0.15	-	-	-
10A	4	2566	S	S	S	S	I	S	**R**	S	S	S	S	**R**	**R**	**R**	MDR	128	256	-	0.02	-	/	/
10H **†**	**5**	1726	**R**	**R**	**R**	**R**	**R**	**R**	**R**	**R**	**R**	**R**	**R**	**R**	**R**	S	XDR	*2048*	*512*	*	8.79	+	-	-
27H **†**	**5**	1727	**R**	S	**R**	**R**	**R**	**R**	**R**	**R**	**R**	**R**	**R**	**R**	**R**	**R**	XDR	*2048*	*512*	*	123.89	+	-	-
9H	**5**	1724	**R**	**R**	**R**	**R**	**R**	**R**	**R**	I	**R**	**R**	**R**	**R**	**R**	**R**	XDR	*2048*	*256*	*	0.05	-	-	-
2H **†**	**5**	1724	**R**	**R**	**R**	**R**	**R**	**R**	**R**	I	**R**	**R**	**R**	**R**	**R**	S	XDR	**2048**	**128**	**+**	0.15	-	-	-
28H **†**	**5**	1728	**R**	**R**	**R**	**R**	**R**	**R**	**R**	I	**R**	**R**	**R**	**R**	**R**	S	XDR	**2048**	**8**	**+**	0.03	-	-	-
17H	6	1733	S	S	S	S	**R**	S	S	S	S	S	S	**R**	**R**	**R**	MDR	**2048**	**128**	**+**	0.07	-	ND	ND
11A	7	2567	S	S	S	S	S	S	S	S	I	S	S	**R**	**R**	**R**	MDR	256	256	-	1.01	-	/	/
68H	**8**	2710	**R**	**R**	**R**	**R**	**R**	**R**	**R**	I	**R**	**R**	**R**	**R**	**R**	**R**	XDR	*1024*	*512*	-	2.65	-	+	-
70H	**8**	2716	**R**	**R**	**R**	**R**	**R**	**R**	**R**	I	**R**	**R**	**R**	**R**	**R**	**R**	XDR	*1024*	*512*	-	97.90	+	+	-
63H	**8**	2704	**R**	**R**	**R**	**R**	**R**	**R**	**R**	I	**R**	**R**	**R**	**R**	**R**	**R**	XDR	*2048*	*512*	*	316.39	+	ND	ND
67H	**8**	2710	**R**	**R**	**R**	**R**	**R**	**R**	**R**	R	**R**	**R**	**R**	**R**	**R**	**R**	**PDR**	*1024*	*512*	-	1041.74	+	+	-
72H	**8**	2731	**R**	**R**	**R**	**R**	**R**	**R**	**R**	R	**R**	**R**	**R**	**R**	**R**	**R**	**PDR**	*2048*	*1024*	-	8.81	+	+	-
69H	9	2713	**R**	**R**	**R**	**R**	**R**	**R**	**R**	I	**R**	**R**	**R**	**R**	**R**	**R**	XDR	*1024*	*512*	-	2.13	-	-	+
73H	10	2732	S	S	S	**R**	**R**	S	I	I	**R**	**R**	**R**	**R**	**R**	**R**	MDR	512	512	-	3.17	+	-	-
2A	11	2249	S	S	S	S	S	S	S	S	S	S	S	**R**	**R**	**R**	MDR	128	128	-	0.48	-	/	/
9A	11	2565	S	S	S	S	R	S	S	S	I	**R**	I	**R**	**R**	**R**	MDR	256	256	-	2.76	-	-	-
71H	**12**	2559	**R**	**R**	**R**	**R**	**R**	**R**	**R**	**R**	S	**R**	**R**	**R**	**R**	**R**	XDR	1024	1024	-	231.70	+	-	+
65H	**12**	233	**R**	**R**	**R**	**R**	**R**	**R**	**R**	**R**	S	**R**	**R**	**R**	**R**	**R**	XDR	*2048*	*1024*	-	1485.21	+	-	+
75H	**12**	233	**R**	**R**	**R**	**R**	**R**	**R**	**R**	I	S	**R**	**R**	**R**	**R**	**R**	XDR	*2048*	*1024*	-	2.44	-	-	+
76H	**12**	233	**R**	**R**	**R**	**R**	**R**	**R**	**R**	**R**	S	**R**	**R**	**R**	**R**	**R**	XDR	*2048*	*1024*	-	100.34	+	ND	ND
57H	**12**	233	**R**	**R**	**R**	**R**	**R**	**R**	**R**	**R**	S	**R**	**R**	**R**	**R**	**R**	XDR	*2048*	*512*	*	127.01	+	-	+
59H	**12**	233	**R**	**R**	**R**	**R**	**R**	**R**	**R**	**R**	S	**R**	**R**	**R**	**R**	**R**	XDR	*2048*	*512*	*	1.47	-	-	+
77H	**12**	233	**R**	**R**	**R**	**R**	**R**	**R**	**R**	**R**	S	**R**	**R**	**R**	**R**	**R**	XDR	*2048*	*512*	*	44.75	+	-	+
74H	**12**	2560	**R**	**R**	**R**	**R**	**R**	**R**	**R**	**R**	I	**R**	**R**	**R**	**R**	**R**	XDR	*2048*	*1024*	-	50.45	+	-	+
66H **†**	**12**	2559	**R**	**R**	**R**	**R**	**R**	**R**	**R**	**R**	S	**R**	**R**	**R**	**R**	**R**	XDR	*2048*	*1024*	-	4.43	+	-	+
1A	13	2561	S	S	S	S	**R**	S	**R**	I	**R**	S	S	**R**	**R**	S	MDR	512	512	-	0.83	-	ND	ND
23H	14	1736	S	S	S	S	S	S	S	S	I	I	I	**R**	**R**	**R**	MDR	*128*	*32*	*	30.22	+	/	/
24H	14	1736	S	S	S	S	I	S	S	S	S	S	S	**R**	**R**	**R**	MDR	*128*	*16*	*	9.74	+	/	/
61H **†**	15	2557	S	S	S	S	S	S	I	S	S	S	S	**R**	**R**	**R**	MDR	*2048*	*512*	*	90.93	+	/	/
13A	16	2568	S	S	S	S	**R**	S	**R**	S	S	**R**	S	**R**	**R**	S	MDR	128	256	-	8.30	+	+	-
64H	17	2709	**R**	**R**	S	**R**	S	**R**	S	I	I	S	**R**	**R**	**R**	**R**	XDR	*1024*	*256*	*	89.14	+	ND	ND
60H **†**	18	2248	**R**	**R**	S	**R**	**R**	**R**	**R**	I	**R**	I	**R**	**R**	**R**	S	XDR	*2048*	*1024*	-	167.88	+	-	+
62H	18	2558	I	S	**R**	**R**	**R**	**R**	**R**	I	**R**	I	**R**	**R**	**R**	S	XDR	*2048*	*1024*	-	6.63	+	-	+
58H	19	112	**R**	**R**	**R**	I	**R**	**R**	**R**	S	S	S	S	**R**	**R**	**R**	MDR	**1024**	**32**	**+**	4.23	+	-	-
22H	20	1735	S	S	S	S	S	S	S	S	S	S	S	**R**	**R**	**R**	MDR	*2048*	*256*	*	0.55	-	/	/
34H **†**	21	561	S	S	S	S	S	S	S	S	S	S	S	**R**	**R**	**R**	MDR	128	128	-	0.10	-	/	/
13H **†**	21	1737	S	S	S	S	S	S	S	S	S	S	S	**R**	**R**	**R**	MDR	*2048*	*1024*	-	0.31	-	/	/
19H	22	1731	S	S	S	S	S	S	I	S	S	S	R	**R**	**R**	S	MDR	**256**	**32**	**+**	1.48	-	/	/
4A	23	2563	S	S	S	**R**	**R**	S	S	S	**R**	S	S	**R**	**R**	**R**	MDR	*2048*	*1024*	-	0.88	-	ND	ND
21H	24	1734	S	S	S	S	S	S	S	S	S	S	S	**R**	**R**	**R**	MDR	**512**	**128**	**+**	7.00	+	/	/
3A	25	2562	S	S	S	**R**	S	S	S	S	S	S	S	**R**	**R**	**R**	MDR	64	64	-	0.02	-	ND	ND
7A	**26**	2250	S	S	S	S	S	S	S	S	I	S	S	**R**	**R**	S	S	64	128	-	0.04	-	/	/
6A	**26**	540	S	S	S	S	S	S	S	S	S	S	I	**R**	**R**	S	S	128	128	-	0.03	-	/	/
8A	**26**	2251	S	S	S	S	I	S	S	S	S	R	S	**R**	**R**	**R**	MDR	64	64	-	0.03	-	/	/
5A	**26**	2564	S	S	S	S	**R**	S	S	S	S	S	S	**R**	**R**	**R**	MDR	128	128	-	16.73	+	-	-
29H **†**	**26**	1729	**R**	**R**	**R**	**R**	**R**	**R**	**R**	**R**	S	R	R	**R**	**R**	**R**	XDR	*2048*	*256*	*	164.59	+	-	-
14A	**26**	1729	S	S	**R**	S	**R**	S	**R**	I	R	R	S	**R**	**R**	**R**	XDR	*64*	*<8*	*	1.95	-	ND	ND
20H **†**	27	2226	S	S	S	S	S	S	S	S	S	S	S	**R**	**R**	S	S	1024	1024	-	0.07	-	/	/
PAO1		549	S	S	S	S	S	S	S	S	S	S	S	S	**R**	S	S	64	64	-	1.00	-	/	/

**ID**: origin (H: nosocomial strains; A: environmental strains); patient death outcome: **†**

**H:** Haplotype was considered as the DNA sequence from the concatenation of *mexR-nalC-nalD* genes.

**ST:** Sequence Type. The ST1725 (most frequent) and the ST233 (high risk clone) are highlighted.

**Antibiotic categories:** Aminoglycosides (GEN: gentamicin, TOB: tobramycin, AK: amikacin), Carbapenems (IMI: imipenem, MEM: meropenem), Cephalosporins (CAZ: ceftazidime, CPM: cefepime), Penicillins (CB: carbenicillin), Penicillins + β-lactamase inhibitors (P/T: piperacillin-tazobactam), Monobactams (AZT: aztreonam), Fluoroquinolones (CIP: ciprofloxacin, LEV: levofloxacin), Phosphonic Acids (FOS: fosfomycin), Polymyxins (CS: colistin).

**SP:** Susceptibility: S: sensitive, I: intermediate resistant, R: resistant; S: sensitive; MDR: multidrug resistant, XDR: extensively drug resistant, PDR (highlighted): pan drug resistant.

**MexAB-OprM:** MexAB-OprM efflux pump phenotypic detection; carbenicillin (CB), a substrate of the pump, was used as reporter antibiotic; **CB—PaβN**: MIC determined for each strain to CB in the absence of the efflux inhibitor Phe-Arg-β-naphthylamine (PaβN) (50 μg/mL); **CB + PaβN:** MIC determined for each strain to CB in the presence of the efflux inhibitor PaβN (50 μg/mLg/L); ^***a***^**: Elevation of the MIC is caused by the MexAB-OprM efflux pump:** +: MexAB-OprM efflux pump is most likely the cause of the elevation of the MIC (values in bold); *: MexAB-OprM efflux pump is contributing in the elevation of the MIC (values in italics); -: MexAB-OprM efflux pump is unlikely to be the cause of the elevation of the MIC or no elevation of the MIC (underlined values).

**Transcript expression:** *mexA*: transcript expression level; transcript ratio between the target gene *mexA* and the reference gene *rpsL;* ^*b*^: +: mexAB-oprM overtranscription; -: mexAB-oprM basal level.

**Carbapenemase:** S: serine carbapenemase, M: metallo-β-lactamase, +: presence of carbapenemase; ND: not determined; /: meropenem and imipenem sensitive strain; Carbapenemase-producing test was developed in meropenem and/or imipenem resistant strains.

Carbapenemase expression was evaluated in 74 strains confirmed to be meropenem and/or imipenem resistant. The commercial kit β CARBA Test showed invalid results for 15 strains (Table 1). For the remaining 59 strains, carbapenemase typing (serine carbapenemase or metallo-β-lactamase) was conducted (Table 1) and showed that eight strains were positive for serine carbapenemases, and 11 strains were positive for metallo-β-lactamases.

### MexAB-OprM efflux pump contributes to MIC rising behavior

Of the 91 *P*. *aeruginosa* strains, 56.04% [51/91: 9 MDR, 23 XDR, and 19 PDR] demonstrated high phenotypic activity of the MexAB-OprM efflux pump (Table 1). However, the MexAB-OprM efflux pump was considered as the most likely cause of the elevation of the MIC in 52.9% of the strains [27/51: (4 MDR, 9 XDR, and 14 PDR)]. In 47% of the strains [24/51: (5 MDR, 14 XDR, and 5 PDR)] the MexAB-OprM efflux pump was contributing to the elevation of the MIC. Finally, the MexAB-OprM efflux pump was not the cause of the elevation of the MIC in 43.96% of the strains [40/91: (3 S, 14 MDR, 16 XDR,7 PDR)].

Considering the phenotypic positive-strains (Table 1): a significant difference was observed between nosocomial (64.93%, 50/77) and environmental strains (7%, 1/14) (*p*< 0.0001), taking the negative MexAB-OprM efflux pump strains as reference (RRR = 11.07; p = 0.025). In addition, a significant difference was observed between strains classified as PDR (73%; 19/26), XDR (58.97%; 23/39), MDR (39.1%; 9/23) and S (0%;0/3) (p = 0.016), where positive MexAB-OprM efflux pump strains and taking the MDR strains as reference (RRR = 7; p = 008) (Table 1).

### *mexAB-oprM* overtranscription predominates in XDR and PDR strains

Real-time RT-qPCR revealed amplification efficiency values between 102.68% and 110.61% for the *rpsL* and *mexA* genes respectively, with a ratio coefficient of R^2 >^ 0.98. Transcript expression levels for *mexA* showed *mexAB-oprM* overtranscription in 45.05% of the strains [41/91: (8/23 MDR, 22/39 XDR, 11/26 PDR)]; PDR and XDR strains showed the highest values (1041.74-fold and 1485.21-fold) compared to the reference strain *P*. *aeruginosa* PAO1 (Table 1). The Kruskal-Wallis equality of populations rank test showed a statistically significant difference between the transcript expression and the susceptibility profile of the strains (p = 0.0100), (S1 Fig).

### Multilocus sequence typing verifies the diversity of most *P*. *aeruginosa* strains and reveals the emergence of outstanding sequence types

The ST for 58 *P*. *aeruginosa* strains were acquired from the PubMLST data base entry http://pubmlst.org/paeruginosa/ (S1 Table), being the endemic clone ST1725 the most frequent and persistent for over 7 years in the hospital. The remaining 33 strains isolated during 2013–2015 were analyzed here, revealing three new alleles: allele 233 for *aroE*, 147 for *guaA*, and 157 for *mutL;* in addition to 23 new ST that were integrated into the worldwide *P*. *aeruginosa* MLST database (S1 Table). During the same period, six ST233 strains and one ST111 strain (both reported worldwide as high-risk clones) were isolated, with ST111 identified in the environment. Of the 48 ST studied, the environmental strains showed the greatest diversity, with a different ST in each strain (Table 1). The nucleotide and gene diversity were greatest among environmental strains (Pi, 0.0074; Hd, 0.0071), with the greatest diversity observed in the *aroE* and *trpE* genes (Pi, 0.010; Hd, 0.007). Of the 94 SNPs identified, 81 were in hospital strains, with the greatest number seen in the *aroE* (n = 18) and *trpE* (n = 19) genes. Relevant genetic data, including the number of haplotypes, nucleotide diversity, gene diversity, and substitutions identified by MLST genotyping are summarized in Table 2.

**Table 2:** Number of haplotypes, nucleotide diversity, gene diversity and substitutions identified by gene for the genotyping of the *P*. *aeruginosa* strains via MLST.

MLST	H strains (n = 77)	A strains (n = 14)	H + A (n = 91)	*acsA*	*aroE*	*guaA*	*mutL*	*nuoD*	*ppsA*	*trpE*
**Size (nucleotides)**	2882	2882	2882	390	498	373	442	366	370	443
**Number of Haplotypes**	35	14	48	18	12	14	17	9	14	16
**Pi**	0.0067	0.0074	0.0071	0.0099	0.0102	0.0055	0.0036	0.0024	0.007	0.0101
**Eta**	0.0057	0.0071	0.0064	0.0066	0.0071	0.0063	0.0049	0.0054	0.0059	0.0084
**Hd**	0.802	1	0.858	0.769	0.751	0.766	0.678	0.557	0.777	0.779
**S**	81	65	94	13	18	12	11	10	11	19

H: nosocomial strains; A: environmental strains.

Haplotype was considered as the DNA sequence from the concatenated sequences obtained from the Multilocus Sequence Typing (MLST).

Pi: nucleotide diversity; Eta: theta (per site); Hd: gene diversity; S: substitutions.

### SNPs in the regulatory *mexR*, *nalC*, and *nalD* genes show *nalC* gene as the most diverse

It was observed a total of 62 nucleotide substitutions, 49 synonymous and 13 non-synonymous in the *mexR*, *nalC*, and *nalD* genes (S2 Table). The *mexR* gene had 13 synonymous substitutions in 74.7% of the strains (68/91) and three non-synonymous substitutions in 71.42% (65/91) of the strains, with the V_126_E amino acid variation being the most frequent (69.23%; 63/91). The ^268^C→T nonsense substitution, the only substitution encoding a stop codon (Q_90_*) was observed in two strains. The *nalC* gene had 19 synonymous substitutions in 76.92% (70/91) of the strains and nine non-synonymous substitutions in 98.90% (90/91) of the strains, with G_71_E being the most frequent 96.70%; (88/91). Additionally, one strain had a 12-bp deletion from position 105 to 116. Finally, the *nalD* gene had 17 synonymous substitutions in 67.03% of the strains (61/91) and one non-synonymous (A_46_T) in 6.59% of the strains (6/91). The *nalC* gene had the highest number of substitutions.

Haplotype was defined as the DNA sequence of the concatenated *mexR-nalC-nalD* genes. A total of 27 different haplotypes were identified in the 91 strains, including 26 haplotypes with substitutions and one haplotype with a 12-bp deletion (S2 Table and Table 3). The hospital strains showed the largest number of haplotypes (n = 19) (Table 1), while the environmental strains had the greatest diversity in nucleotides (Pi) and genes (Hd) (Pi, 0.00922; Hd, 0.879), with the greatest diversity observed for the *nalC* gene (Pi, 0.01184; Hd, 0.771) (Table 2). We observed a total of 62 SNPs, of which 61 were in hospital strains and most were in the *nalC* gene (n = 28) (S2 Table).

**Table 3:** Genetic variations (haplotypes) identified in the *mexR*, *nalC* and *nalD* repressor genes in *P*. *aeruginosa* strains.

Haplotype	*mexR*	*nalC*	*nalD*
**1**	S_88_S, E_109_E, Q_128_Q, Q_137_Q, **V**_**126**_**E (1)**	A_4_A, S_5_S, A_23_A, I_41_I, R_43_R, G_49_G, E_59_E, S_118_S, Y_137_Y, A_145_A, A_148_A, P_149_P, **G**_**71**_**E, S**_**209**_**R (1)**	L_57_L, L_99_L **(1)**
**2**	S_88_S, E_109_E, Q_128_Q, Q_137_Q, **V**_**126**_**E (1)**	A_4_A, S_5_S, A_23_A, I_41_I, R_43_R, G_49_G, E_59_E, S_118_S, Y_137_Y, A_145_A, A_148_A, P_149_P, **(T**_**35**_△, **T**_**36**_△, **L**_**37**_△, **D**_**38**_△, **M**_**39**_△**), G**_**71**_**E, S**_**209**_**R (2)**	L_57_L, L_99_L **(1)**
**3**	S_88_S, E_109_E, Q_128_Q, Q_137_Q, **V**_**126**_**E (1)**	A_4_A, S_5_S, A_23_A, I_41_I, R_43_R, G_49_G, E_59_E, S_118_S, A_123_A, Y_137_Y, A_145_A, A_148_A, P_149_P, **G**_**71**_**E, D**_**79**_**E, S**_**209**_**R (3)**	K_26_K, L_99_L, R_150_R, P_159_P **(2)**
**4**	V_20_V, E_109_E, Q_128_Q, Q_137_Q, **V**_**126**_**E (1)**	A_4_A, S_5_S, A_23_A, I_41_I, R_43_R, G_49_G, E_59_E, S_118_S, A_123_A, Y_137_Y, A_145_A, A_148_A, P_149_P, **G**_**71**_**E, S**_**209**_**R (4)**	G_45_G **(3)**
**5**	V_20_V, E_109_E, Q_128_Q, Q_137_Q, **V**_**126**_**E (1)**	A_4_A, S_5_S, A_23_A, I_41_I, G_49_G, E_59_E, S_118_S, R_120_R, A_123_A, Y_137_Y, A_145_A, A_148_A, P_149_P, **G**_**71**_**E, E**_**153**_**Q, S**_**209**_**R** **(5)**	C_92_C, L_99_L, I_111_I, D_180_D **(4)**
**6**	V_5_V, N_6_N, P_11_P, T_22_T, R_32_R, S_88_S, E_109_E Q_128_Q, Q_137_Q, **V**_**126**_**E (3)**	A_4_A, S_5_S, A_23_A, I_41_I, G_49_G, E_59_E, S_118_S, Y_137_Y, A_145_A, A_148_A, P_149_P, **G**_**71**_**E, A**_**145**_**V, S**_**209**_**R (6)**	L_57_L, C_92_C, L_99_L, D_180_D **(5)**
**7**	V_5_V, N_6_N, P_11_P, R_32_R, D_56_D, S_88_S, E_109_E, Q_128_Q, Q_137_Q, **V**_**126**_**E (4)**	**G**_**71**_ **E, S**_**209**_ **R (7)**	**(6)**
**8**	V_5_V, N_6_N, P_11_P, R_32_R, S_88_S, E_109_E, Q_128_Q, Q_137_Q, **V**_**126**_**E (5)**	A_23_A, **G**_**71**_**E, S**_**209**_**R** **(8)**	**A**_**46**_ **T (7)**
**9**	V_5_V, N_6_N, P_11_P, R_32_R, S_88_S, E_109_E, Q_128_Q, Q_137_Q, **V**_**126**_**E (5)**	A_23_A, **G**_**71**_**E** **(9)**	**A**_**46**_ **T (7)**
**10**	S_88_S, E_109_E, Q_128_Q, Q_137_Q, **L**_**57**_**P, V**_**126**_**E** **(6)**	**G**_**71**_ **E, S**_**209**_ **R (7)**	**(6)**
**11**	**(7)**	**G**_**71**_ **E, S**_**209**_ **R (7)**	**(6)**
**12**	**(7)**	**G**_**71**_ **E, E**_**153**_ **D, A**_**186**_ **T (10)**	**(6)**
**13**	**(7)**	**(11)**	**(6)**
**14**	**(7)**	**A**_**44**_ **T (12)**	**(6)**
**15**	V_126_V, Q_137_Q. **(8)**	**G**_**71**_ **E (13)**	**(6)**
**16**	L_67_L **(9)**	**G**_**71**_ **E, S**_**209**_ **R (7)**	**(6)**
**17**	L_67_L **(9)**	**G**_**71**_ **E, S**_**209**_ **R (7)**	A_40_A. **(8)**
**18**	**Q**_**90**_ * (10)	**G**_**71**_ **E, S**_**209**_ **R (7)**	**(6)**
**19**	D_56_D **(11)**	T_86_T, V_147_V, P_149_P, **G**_**71**_**E (14)**	**(6)**
**20**	**(7)**	T_86_T, V_147_V, P_149_P, **G**_**71**_**E, S**_**209**_**R (15)**	F_51_F, L_57_L, L_99_L. **(9)**
**21**	**(7)**	A_23_A, **G**_**71**_**E, S**_**209**_**R (8)**	**(6)**
**22**	D_56_D **(11)**	A_23_A, **G**_**71**_**E, S**_**209**_**R (8)**	D_185_D **(10)**
**23**	**(7)**	A_23_A, **G**_**71**_**E (9)**	**(6)**
**24**	**(7)**	A_4_A, S_5_S, **G**_**71**_**E, S**_**209**_**R (16)**	A_168_A. **(11)**
**25**	**(7)**	A_4_A, S_5_S, A_23_A, I_41_I, R_43_R, G_49_G, E_59_E, S_118_S, A_123_A, Y_137_Y, A_145_A, A_148_A, P_149_P, **G**_**71**_**E, S**_**209**_**R (4)**	R_150_R, P_159_P, D_185_D **(12)**
**26**	V_20_V, E_109_E, Q_128_Q, Q_137_Q, **V**_**126**_**E** **(2)**	A_4_A, S_5_S, A_23_A, I_41_I, G_49_G, E_59_E, F_62_F, F_98_F, S_118_S, Y_137_Y, A_145_A, A_148_A, P_149_P, A_186_A, **G**_**71**_**E, A**_**145**_**V, S**_**209**_**R (17)**	K_26_K, A_55_A, T_101_T, R_150_R, P_159_P **(13)**
**27**	**(7)**	A_4_A, S_5_S, A_23_A, I_41_I, G_49_G, E_59_E, S_118_S, R_120_R, A_123_A, Y_137_Y, A_145_A, A_148_A, P_149_P, **G**_**71**_**E, E**_**153**_**Q, S**_**209**_**R (5)**	S_77_S, L_99_L **(14)**
**Number of haplotypes**	11	17	14
**Pi**	0.00745	0.01184	0.00376
**Eta**	0.00709	0.00906	0.00585
**Hd**	0.718	0.771	0.723
**S**	16	28	18

First letter indicates reference strain (*P*. *aeruginosa* PAO1) amino acid, the number indicates the amino acid position where the change occurs, and the second letter indicates the amino acid that substitutes the original amino acid. The symbol △means nucleotide deletion. The * means stop codon. Amino acid variation: A: alanine, C: cysteine, D: aspartic acid, E: glutamic acid, F: phenylalanine, G: glycine, H: histidine, I: isoleucine, K: lysine, L: leucine, M: methionine, N: asparagine, P: proline, Q: glutamine, R: Arginine, S: serine, T: threonine, V: valine, W: tryptophan, Y: tyrosine. In bold are non-synonymous substitutions. Pi: nucleotide diversity, Eta: theta (per site), Hd: gene diversity, S: nucleotide substitutions. In haplotype 13 (no SNPs identified), the *mexR*, *nalC* and *nalD* repressor genes of the MexAB-OprM efflux pump sequences were identical to those of *P*. *aeruginosa* PAO1. In parenthesis, the number of haplotypes.

### Phylogenetic analysis arranges *P*. *aeruginosa* strains into genetic complexes that share the same characteristics including *mexR-nalC-nalD* haplotypes

**Fig 1:**
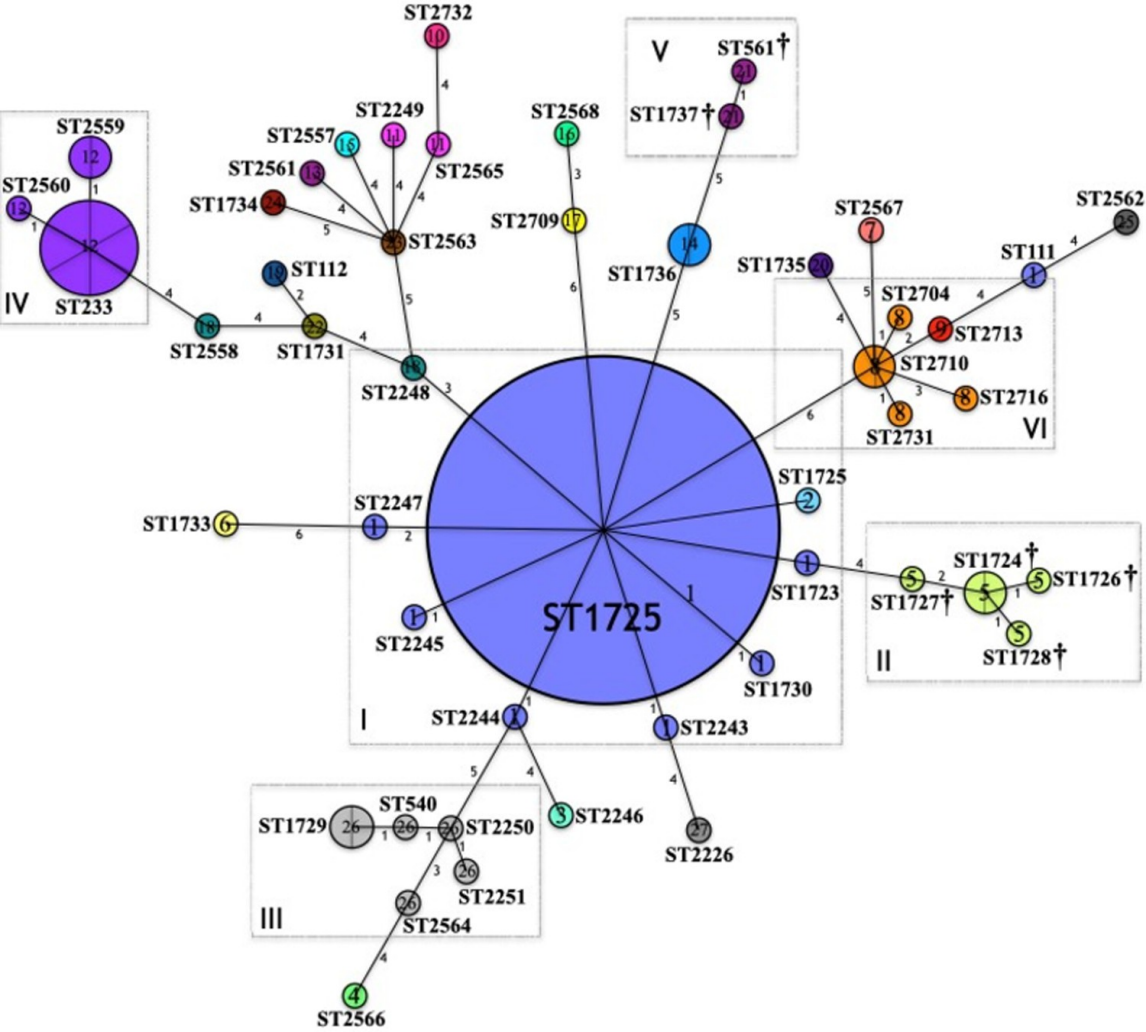
Phylogenetic network based on the MLST genotyping of the *P*. *aeruginosa* strains (ST/haplotypes). Phylogenetic relationships, evolutionary history, clonal complexes, and relationship between the *mexR-nalC-nalD* haplotypes and the STs are shown. *P*. *aeruginosa* isolates (n = 91, 48 STs, 27 mexR-nalC-nalD haplotypes). Circles represent sequence types (STs); Circumference is based on ST frequency; Two or more strains with the same ST are depicted as fractions in each circle (ST1725, n = 34 strains); Lines connect locus variants; Numbers indicate the number of locus variants among the connected STs. Clonal complexes (CC) formed are highlighted in rectangles and described as I, II, III, IV, V and, VI. STs not grouped into a CC are considered singletons (>3 locus variants with other STs). Numbers inside the circles (1–27) corresponds with the mexR-nalC-nalD haplotype and are colored differently. †: Fatal patient outcome.

**Fig 2:**
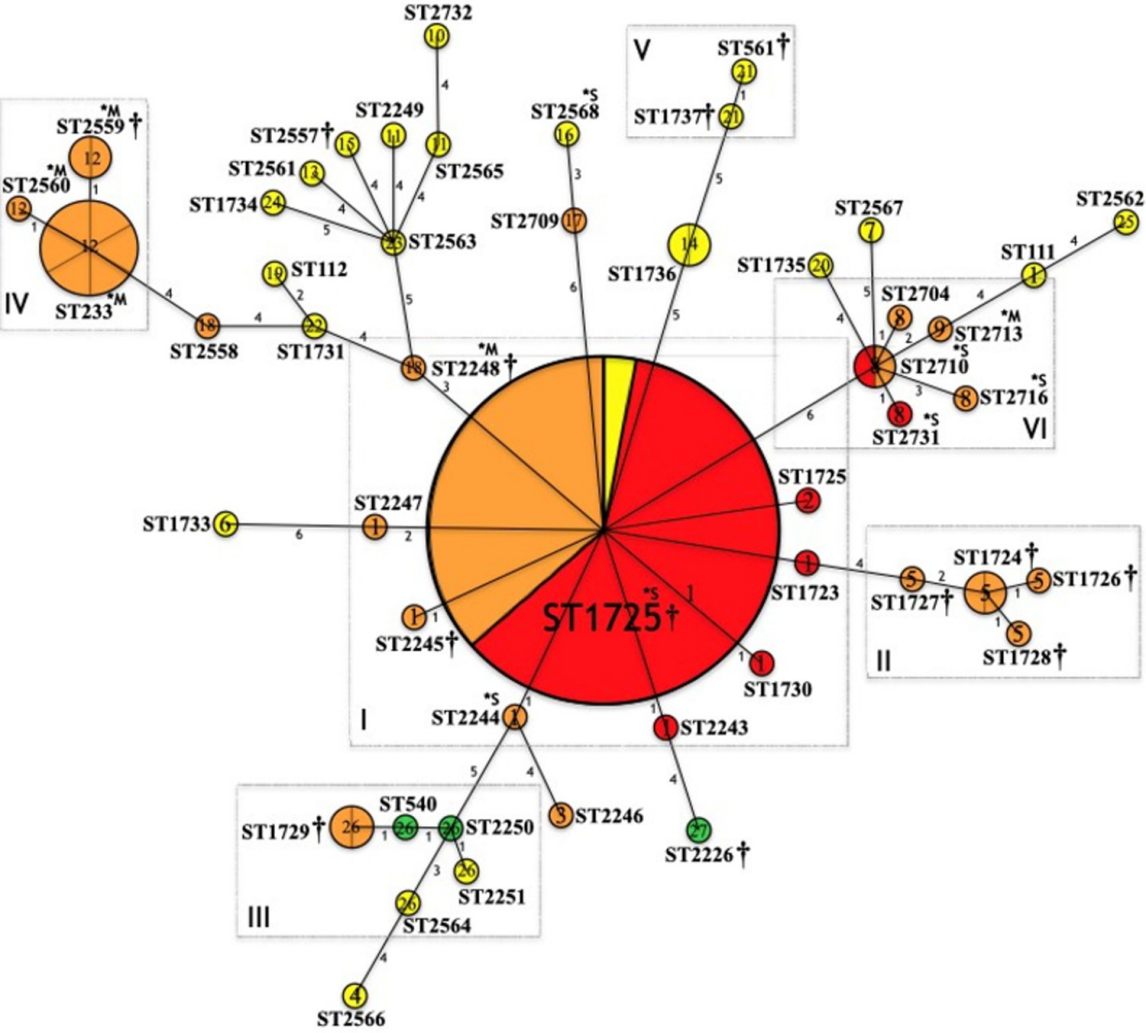
Phylogenetic network based on the MLST genotyping of the *P*. *aeruginosa* strains (ST/haplotypes/resistance). Relationship between the *mexR-nalC-nalD* haplotypes, the STs and, the susceptibility profiles are shown. *P*. *aeruginosa* isolates (n = 91, 48 STs, 27 mexR-nalC-nalD haplotypes). Circles represent sequence types (STs); Circumference is based on ST frequency; Two or more strains with the same ST, including ST1725 are depicted as fractions in each circle (n = 34 strains); Lines connect locus variants; Numbers indicate the number of locus variants among the connected STs. Clonal complexes (CC) formed are highlighted in rectangles and described as I, II, III, IV, V and, VI. STs not grouped into a CC are considered singletons (>3 locus variants with other STs). Number inside the circles (1–27) corresponds with the mexR-nalC-nalD haplotype and are colored differently. †: Fatal patient outcome. Susceptibility profiles: S (green), sensitive; MDR (yellow), multidrug resistant; XDR (orange), extensively drug resistant; PDR (red), pan drug resistant. *S: Serine carbapenemase; M*: Metallo-β-lactamase.

The phylogenetic network based on the MLST genotyping (ST) of the *P*. *aeruginosa* strains is shown in Figs 1 and 2. Although both figures refer to the same phylogenetic network, different information is highlighted. The phylogenetic relationships, evolutionary relationship, clonal complexes identified in the 48 STs and the 27 *mexR-nalC-nalD* haplotypes are shown in Fig 1; in Fig 2, the relationship between the 27 identified *mexR-nalC-nalD* haplotypes, the 48 STs, the susceptibility profiles, and production of carbapenemases are depicted. The data indicated six important clonal complexes (CC); in each complex, all STs have a close phylogenetic relationship as follows:

#### Complex I

Includes strains ST1725 as the most prevalent (n = 34), followed by ST1723, ST1730, ST2243, ST2244, ST2245, ST2247 and ST2248 (n = 1 strain each), all nosocomial origin (Fig 1). The ST1725 was identified by the BURST analysis as the potential Ancestral Type (AT) of this clonal complex (Fig 1). Globally reported data in the PubMLST *P*. *aeruginosa* database describe this complex as part of the CC309.

Haplotype 1 was highly associated with Complex I compared to other haplotypes and taking the singletons STs as reference (RRR = 409.53; p = 0.000). Haplotype 1 was identified with high frequency (n = 40); all ST1725 strains presented this haplotype except one isolate classified as haplotype 2 because of a deletion (△^105–116^). ST111, a high-risk clone, also presented this haplotype (although it appears to be phylogenetically distant from CC1). Haplotype 1 was identified in strains of different ST, suggesting that these substitutions are specific to phylogenetically related ST (Fig 1).

In addition, this complex showed MDR (4.76% MDR, 38.10% XDR and 57.14% PDR) (Fig 2). In 51.22% of the strains [21/41: (14 PDR and 7 XDR)] the MexAB-OprM efflux pump was the most likely cause of the elevation of the MIC, and in 21.95% of the strains [9/41: (1 MDR, 3 XDR, 5 PDR)] the pump was contributing in the elevation of the MIC; in addition, 39.02% of *mexAB-oprM* overtranscription was detected in the strains [16/41: (8 XDR, 8 PDR)] (Table 1). Serine carbapenemases were identified in three strains (ST1725 and ST2244) and two strains produced metallo-β-lactamases (ST2248) (Table 1). Association between haplotypes and high-drug resistance may be due to potential relationship between the ST and *mexR-nalC-nalD* haplotypes, as resistance is highly related to specific STs (Table 1 and Fig 2).

#### Complex II

Includes ST1724, ST1726, ST1728 and ST1727, all nosocomial origin with the ST1724 identified as the AT (Fig 1). This complex form part of the globally CC235. All STs that make up this complex presented haplotype 5 and showed XDR (Figs 1 and 2). The relationship between this haplotype and fatal patient outcomes was remarkable. Complex II was associated with death when compared to the singletons STs (RRR = 40.01; p = 0.006), to complex I (RRR = 23.34; p = 0.009), and to complex 4 (RRR = 32.01; p = 0.025). The high-drug resistance exhibited by these strains could be in part attributed to the MexAB-OprM efflux pump activity; in two strains (2/5), efflux pump activity was the most likely cause of the elevation of the MIC, and in three strains (3/5) efflux pump was contributing to the elevation of the MIC. *mexAB-oprM* overtranscription was detected in two strains (Table 1 and Fig 2).

#### Complex III

Includes ST1729, ST540, ST2250, and ST2251, all environmental origin except for one XDR ST1729 strain that was associated with a fatal outcome in 2009 (Figs 1 and 2). No AT was identified within these STs; however, they are part of the global CC253. All STs that make up this complex presented haplotype 26 and showed variations in antimicrobial susceptibility (S, MDR, and XDR) (Figs 1 and 2). In 2/6 strains, efflux pump activity was contributing to the elevation of the MIC. *mexAB-oprM* overtranscription was detected in two strains (1 MDR, 1 XDR) (Table 1).

#### Complex IV

Includes ST2559, ST2560, and ST233 (n = 6), all nosocomial origin (Fig 1). ST233 is considered the AT of this complex, conforming the CC233 worldwide. All STs that make up this complex presented haplotype 12, were XDR and produced metallo-β-lactamases (Figs 1 and 2). In three strains (3/9), the activity of the pump was contributing to the elevation of the MIC. *mexAB-oprM* overtranscription was detected in seven strains (Table 1).

#### Complex V

Includes ST1737 and ST561, both of nosocomial origin and being part of the CC245 worldwide. Both STs that make up this complex presented haplotype 21 and are possibly associated with fatal patient outcomes (Fig 1). This complex presented MDR and the MexAB-OprM efflux pump was not the cause of the elevation of the MIC. *mexAB-oprM* overtranscription was not detected (Table 1 and Fig 2).

#### Complex VI

Includes ST2710, ST2704, ST2713, ST2716, and ST2731. All these STs were of nosocomial origin with ST2710 identified as the AT but no global CC identified. All STs that make up this complex presented haplotype 8, except for a ST2313 strain that presented haplotype 9, being the only difference between these haplotypes a SNP in the *nalC* gene S_209_R (Fig 1 and Table 3). This complex showed MDR (66.66% XDR and 33.33% PDR) (Fig 2). Only in one strain, the MexAB-OprM efflux pump was contributing to resistance; in the remaining strains, the pump was not the cause of the elevation of the MIC. Haplotype 8 strains produced serine carbapenemases and the haplotype 9 strain produced metallo-β-lactamases. *mexAB-oprM* overtranscription was detected in four strains (2 XDR, 2 PDR) (Table 1).

The rest of the STs are considered singletons since more than 3 locus variants are noticed between them and other STs. The remaining 19 *mexR-nalC-nalD* haplotypes were identified in the STs considered singletons (Fig 1). Most of these STs were MDR and only one strain (ST2568) produced serine carbapenemases (Table 1 and Fig 2).

Close phylogenetic relationship between CCI and CCII is evident, while the CCIV is the most distant complex. However, all CCs appear to be related somehow to the CCI (Fig 1).

**Fig 3:**
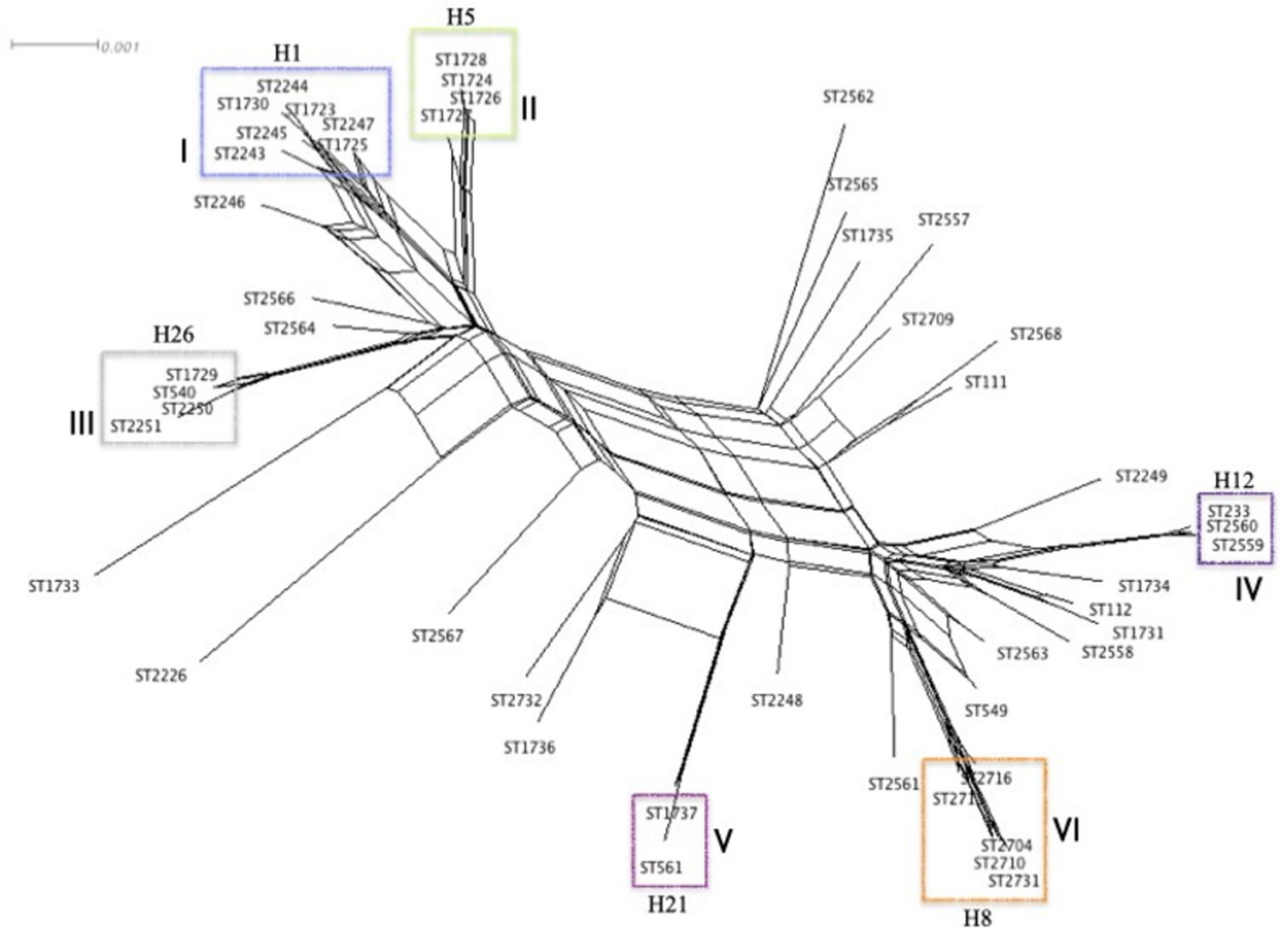
Neighbor-net graph based on the MLST genotyping of the *P*. *aeruginosa* strains. *P*. *aeruginosa* isolates (48 STs). Rectangular boxes represent the high probability of extensive homologous recombination. The PHI test detected statistically significant evidence of recombination (*p =* 0.0). Clonal complexes (CC) formed are highlighted in rectangles and described as (I, II, III, IV, V, and VI). The *mexR-nalC-nalD* haplotype of each CC is shown.

In addition, the six clonal complexes previously identified in the phylogenetic networks stand out as groups of highly maintained STs in the neighbor-net graph (Fig 3). The presence of rectangular boxes in the network represents the high probability of extensive homologous recombination, which was corroborated with the PHI test that revealed statistically significant recombination events (p<0.05). According to the neighbor-net graph, CCII (Haplotype 5) and CCIII (Haplotype 26) appear to be closely associated with the CC1 (Haplotype 1), but distant from CCIV (Haplotype 12) and CCVI (Haplotype 8) which are close and are related to production of carbapenemases, indicating that despite the highly recombinant nature of *P*. *aeruginosa*, some substitutions are highly maintained among the strains (CCs). This close relationship between specific ST that differs little in sequence (CCs) is troubling, as it raises the possibility of the eventual selection of an efficient high-risk clone with high dissemination capacity.

### Principal component analysis and statistical analysis reveal association between the ST and the *mexR-nalC-nalD* haplotype

**Fig 4:**
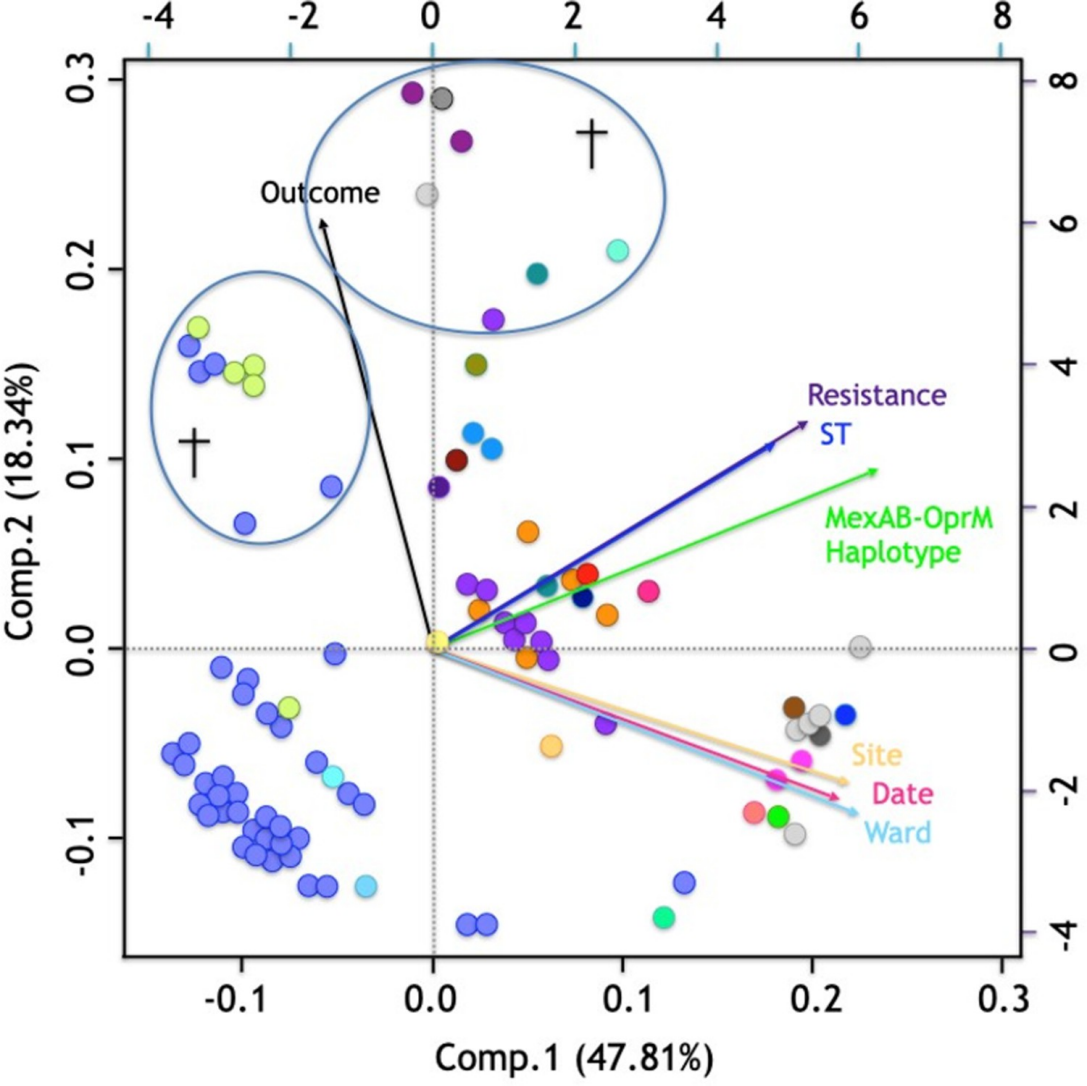
Principal component analysis of the 91 *P*. *aeruginosa* strains. *P*. *aeruginosa* isolates (n = 91). Each strain is represented by a colored dot according to *mexR-nalC-nalD* haplotype (Fig 1). †: Fatal patient outcome. Variables included: Outcome (black vector): living, died; Resistance (purple vector): sensitive (S), multi-drug resistant (MDR), extensively drug resistant (XDR), and pan drug resistant (PDR); ST (blue vector) (n = 48); MexAB-OprM haplotype (*mexR-nalC-nalD*) (green vector) (n = 27); Isolation site (yellow vector): hospital, environmental; Date (pink vector): 2007–2013; Hospital ward (aqua vector): Stx, surgical therapy; E, emergency room; N, neurology; PICU, pediatric intensive care unit; P, pediatrics; S, surgery; Neph, nephrology; O, oncology; It, internal therapy; C, cardiology; NICU, neonatal intensive care unit; Ne, necropsy; IMed, internal medicine.

PCA analysis showed a strong relationship between resistance and ST (Fig 4). The first two principal components of the analysis explained 66.15% of the observed variation, specifically the first component explained 47.81% and the second 18.34% (Fig 4). PCA also revealed ST233 strains and 41.18% of strains with other STs were XDR; of the ST1725 strains, 61.76% were PDR and 35.29% were XDR (*p*< 0.0001); ST1725 was the main ST associated with XDR when compared to the environmental STs and taking the MDR strains as reference (RRR = 65.98; p = 0.001); and with PDR when compared to the hospital STs and taking the MDR strains as reference (RRR = 46.19; p = 0.001).

In addition, the MexAB-OprM haplotype (*mexR-nalC-nalD*) were associated with the ST (*p*< 0.0001) and resistance (*p*< 0.0001) (this last one due to the association between ST and resistance).

PCA showed a relationship between the strain isolation site, isolation date, and hospital ward. However, these variables were inversely proportional to ST and haplotype. On the other hand, the ST and haplotype variables showed closeness and the same direction. The outcome variable (fatal outcome) was inversely proportional to all analyzed variables, although an association was observed between haplotype 5 strains (*mexR-nalC-nalD*) (Fig 4, green dots), haplotype 1 strains (blue dots), and fatal outcomes. Fatal outcomes were observed in 12.5% of haplotype 1 strains, 80% of haplotype 5 strains, 11.11% of haplotype 12 strains, and 16.67% of haplotype 26 strains (*p* = 0.051) (Figs 1 and 4, see haplotype colors). However, it should note that patients’ underlying conditions were not considered in this study.

Statistical analysis of the relationship between the SNPs in the *mexR-nalC-nalD* genes, the sequence type, and patient death outcome gave the following results:

1. **Sequence type (ST):** It was observed that phylogenetically related sequence types presented equal or similar *mexR-nalC-nalD* haplotypes (p <0.05) (Fig 1, see color by haplotype and phylogenetic relationships between STs; S3 Table, see similar *mexR-nalC-nalD* haplotypes).
2. **Patient death outcomes:** It was observed that seven SNPs were associated with patient death outcomes (all identified in haplotype 5 strains), including one in the *mexR* gene (V_20_V), three in the *nalC* gene (R_120_R, A_123_A, and E_153_Q), and three in the *nalD* gene (C_92_C, I_111_I, and D_180_D) (*p*< 0.05) (S3 Table). However, it should note that patients’ underlying conditions were not considered in this study.

## Discussion

This study identifies SNPs in the regulatory *mexR*, *nalC*, *and nalD* genes of the MexAB-OprM efflux pump in clinical and environmental isolates of *P*. *aeruginosa* (including high-risk clones) to discern whether these changes are associated with the pressure exerted by environmental conditions or are mainly related to genetic lineages.

*P*. *aeruginosa* has a great capacity to resist adverse environments, as evidenced by its nosocomial survival. Worldwide, different studies have reported between 27.6 and 71.4% resistant isolates [6, 24, 51]. We observed a high fraction of PDR (33.7%) and XDR (78%) in strains isolated from a hospital setting, while those from environmental settings were MDR (78%). In our work and worldwide, the worrisome observation has been made that colistin resistant strains are present in both nosocomial and other environments while colistin is the last therapeutic option for MDR and XDR isolates [52, 53]. Dößelmann *et al*. (2017) warn about the rapid acquisition of colistin resistance after 10 and 20 days of exposure [53]. The presence of antibiotics or heavy metals in the environment induces MDR and even XDR environmental strains. The finding of resistant bacteria in the environment is attributed to the discharge of antibiotics into waste waters [54] and in industrial waste and the misuse of antibiotics as a preventive measure in livestock and fish farms [4]. Regarding heavy metals, contamination could be accomplished to destruction of built; waste disposal; soil and rock erosion; and industrial and agricultural practices. Sub-inhibitory levels of heavy metals can produce selective pressure on bacterial populations contributing to MDR through co-selection mechanisms (co-resistance, cross-resistance, and co-regulatory resistance) [55–58]. These factors may explain our observed high resistance to numerous antibiotics (including fosfomycin) in environmental and clinical strains, which may interact in nature.

We identified a total of 48 different ST of *P*. *aeruginosa* strains of nosocomial and environmental origin (S1 Table). As expected, all the environmental strains had a different ST, representing great diversity and indicating a non-clonal population structure. In contrast, the nosocomial strains had a non-clonal population structure; however, the emergence of highly successful epidemic clones was evident [53] (ST1725 and ST233), as previously described by Aguilar-Rodea *et al*. [6].

Among the nosocomial strains, ST1725 stands out for its high frequency and multidrug resistance (PDR, 21 strains; XDR, 12 strains; MDR, 1 strain). This endemic clone (Complex I) prevailed for over seven years (2007–2013) in the institution. Similarly, we identified six *P*. *aeruginosa* nosocomial strains as ST233 (Complex 4), all were XDR. Although ST233 has been previously reported worldwide as a high-risk clone resistant to AZT, there are no records of colistin resistant strains or even XDR or PDR strains [59–63]. ST233 is a risk for patients in Mexico, as these strains were resistant to colistin and only sensitive to AZT. The AZT susceptibility of our analyzed strains may result from the lack of commercial use of this antibiotic in Mexico due to restrictions. In the United States (Northeast, Ohio), where AZT use is free of such restrictions, AZT-resistant ST233 strains have been reported [60].

We also identified ST111 (Clomplex I) in a MDR environmental strain collected from a water source. ST111 is considered a high-risk clone identified in Croatia and France with MDR association [64–66]. Occasionally, *P*. *aeruginosa* strains migrate from the environment and cause animal or human infections [26, 67]. For this reason, this type of ST is considered highly dangerous and should be kept under surveillance.

MDR *P*. *aeruginosa* strains likely result from several factors. The involvement of overexpression of efflux pumps has gained recognition, particularly the MexAB-OprM pump for its constitutive expression and attribution of resistance to most antibiotics [12–16]. Our results corroborate the relationship between the MDR of a given *P*. *aeruginosa* strain and its MexAB-OprM efflux pump activity, principally in PDR strains (73%). Efflux pump activity was also higher in strains ST1725 and ST233, compared with other STs (*p* *≤*0.0001). Similar results were reported by Arabestani *et al*., (2015), who observed a significant positive correlation between pump activity and antimicrobial resistance [68]. Ozer *et al*., (2012) reported a consistent positive correlation between *mexAB-oprM* transcripts and multidrug resistance in 50 *P*. *aeruginosa* clinical strains [69]. In our study, of the *P*. *aeruginosa* strains that showed MexAB-OprM activity (phenotypic positive-strains, 56.04%), only in 52.9% the efflux pump was the most likely cause of resistance, in the remaining 47% of the strains the efflux pump was contributing to the resistance; *p* overtranscription was detected in 45.05% of the strains; the correlation between both methodologies was 52.75%; however, *mexA* transcript was detected in all the analyzed strains, verifying the MexAB-OprM efflux pump basal level expression. Similarly, Goli et al. (2018) reported a correlation of 66.6% and 68% between the results of MICs with PAβN and *mexB* gene overtranscription in clinical strains [19]. Other authors report that the high correlation between phenotypic and genotypic methodologies was only detected in reference strains or highly characterized laboratory strains, not in clinical isolates where diverse phenotypes were shown due to diverse genetic backgrounds [70–72]. Highlighting the difficult interpretation of phenotypic data due to the co-expression of resistance mechanisms other than efflux (enzymes, plasmid-encoded b-lactamases, mutations, class 1 integrons), and by the selection pressure exerted by treatment with various antibiotics on the patient; arguing that genotypic methods do not necessarily provide data on the final expression of the gene product and its functionality [19, 70, 71]. Molecular mechanisms such as post-transcriptional control of the protein translation rate, half-lives of proteins or mRNAs, RNA stabilization mechanism, and the molecular association of the protein products of expressed genes must be considerate [71, 73]. Additionally, these regulatory mechanisms of transcription, translation, and proteolysis are highly affected by the genetic heterogeneity in clinical isolates, which can lead to discrepancies [71]. In this study, both methodologies demonstrated that although the MexAB-OprM pump shows positive activity and contributes to resistance, this is not the only mechanism of resistance in these strains [18].

SNPs in MexAB-OprM regulators, which are mainly attributed to environmental conditions, can impair their function favoring *mexAB-oprM* overtranscription expression [10, 23, 24]. Recent studies have identified point mutations in the *mexR*, *nalC*, and *nalD* repressor genes of the MexAB-OprM efflux pump [24, 27]; however, it is unknown whether STs or high-risk clones of different origins present the same substitutions in these repressors regardless their genetic lineage. Here, we observed 27 different haplotypes in the three repressor genes. Regarding the *mexR* gene three non-synonymous substitutions were identified in 71.42% of the strains, with the V_126_E amino acid variation being the most frequent, agreeing with previous studies [27]. The ^268^C→T nonsense substitution was the only change that encoded a stop codon (Q_90_*) and was observed in two strains (haplotype 18). Finally, the ^170^T→C nonsense substitution produced the amino acid variation L_57_P in the strain identified as 73H. Nevertheless, when the preliminary modeling of MexR was performed, apparently no effect was noticed on the structure of the protein when considering the amino acid variations V_126_E and Q_90_*; however, a considerable conformational change was observed when modeling the amino acid variation L_57_P, but further studies are needed to know its effect on the final structure of the protein. Most of the non-synonymous substitutions were found in the *nalC* gene, as seen in 98.90% of the strains (90/91). The most frequent substitution was G_71_E (96.70%; 88/91) as previously described by Horna *et al* (2018) [27]. In addition, a 12-bp deletion was identified in one strain (S2 Table and Table 3). Another study also identifies the *nalC* gene as the main site of nucleotide substitutions target, reporting relevant changes (non-synonymous substitutions) in 87% of nosocomial isolates as well as some deletions [27]. Most of the substitutions reported in that study differ from those we observed (Table 3). Regarding the *nalD* gene, the only non-synonymous substitution identified (A_46_T) was previously reported by Suresh et al (2018) [24]. A crystallography study of the NalD protein demonstrated 66.5% similarity and 32.5% identity to the TetR family protein (TtgR), with different binding ligands with antibiotics such as novobiocin (N129 and H167), in addition, the authors also reported that mutations in F175 and N176 alanine interferes in the binding of NalD to its promoter [74]; however, further studies are needed to know the effect of these changes on the final structure of NalC and NalD proteins, standing out that the crystallized structure for NalC has not been reported.

It has been reported that genetic variations as stop codons, frameshifts or deletions lead to loss of functionality of the repressor genes and may contribute to the *mexAB-oprM* over transcription [75] with a consequent increase in bacterial resistance [10, 23, 24]; however, in our study, same haplotypes (SNPs) lead to different MexAB-OprM efflux pump behavior and resistance. The 12-bp deletion identified in the *nalC* gene, could be one of the causes of the PDR in the haplotype 2 strain due to the malfunction of this repressor (*mexAB-oprM* overtranscription). In the case of the stop codon identified in the *mexR* gene, *mexAB-oprM* overexpression was detected, in addition to the production of metallo-β-lactamases, both mechanisms could explain the XDR shown by these strains. However, in some cases correlation between *mexA* transcript level and haplotype was not evident, may be because in these, other newly discovered regulators as MdrR1, MdrR2, BrlR, CpxR, ArmR and PA3225 could be potentially involved in the transcription of the pump genes [76].

Specific haplotypes were identified within the different complexes, suggesting that these substitutions are highly maintained within phylogenetically related ST no matter what environment they came from. In addition, high-drug resistance exhibited by some of these complexes, could be mostly attributed to the MexAB-OprM activity, however different behavior of the pump (*mexA* transcript expression) was noticed within strains with the same haplotype (for example: haplotypes 8 and 12 which correlate closely with XDR and PDR profiles, mexAB-oprM overtranscription in the 80% and 77.8% of the strains (respectively) was identified), suggesting that the association between haplotypes and high-drug resistance also may be due to potential relationship between the ST and *mexR-nalC-nalD* haplotypes, as resistance is highly related to specific STs.

As haplotype 12 was identified in all ST233 (high risk clones), the sequences (*mexR-nalC-nalD*) detected in this work were compared with six complete genome sequences reported for ST233 strains around the world [36, 77], the finding show a 100% of similarity in the SNPs present in the MexAB-OprM efflux pump regulators which are maintained in *P*. *aeruginosa* genetic lineages regardless the strains origin. In addition, for these strains, the resistance could also be explained by other mechanisms, as explained by Correa *et al*. [8], the presence of carbapenemases stands out in haplotype 12 strains, as also described by other authors in ST233 strains worldwide [5]. Interestingly, the carbapenemase types differ between strains of haplotype 8 (serine carbapenemases) and those of haplotype 12 (metallo-β-lactamases) [5].

## Conclusions

*Pseudomonas aeruginosa* is a highly recombinant microorganism with a great population diversity; however, core genome of this specie is highly conserved, there are genes that despite having a high selection pressure are conserved within genetic lineages, such as the housekeeping genes. For its part, the constitutive MexAB-OprM efflux pump which is related to resistance to antibiotics and virulence of the strains is regulated by three repressors genes. In this work it was demonstrated that nucleotide substitutions in the *mexR*, *nalC* and *nalD* genes are highly maintained in phylogenetically related strains despite the selection pressure exerted by the indiscriminate use of antibiotics. A clear example is the ST233 reported in other parts of the world, in which same mutations present in its repressors could be observed regardless of the geographic location of isolation.

### Nucleotide sequence accession numbers

The nucleotide sequences obtained in this study were deposited in the GenBank database under the following accession numbers: *mexR* gene sequences, MT188163–MT188173; *nalC* gene sequences, MT188174–MT188190; *nalD* gene sequences, MT188191–MT188204.

Accession numbers for the *Pseudomonas aeruginosa* isolates used in this work are available at the public database for molecular typing: PubMLST.org. (See S1 Table) and CDC & FDA Antibiotic Resistance (AR) Isolate Bank (See S3 Table).

## Supporting information

S1 Fig mexAB-oprM transcript expression vs susceptibility profile of the *P*. *aeruginosa* strains. Transcript expression Ratio between the target gene *mexA* and the reference gene rpsL. A: Strains exhibiting mexAB-oprM basal level; B: Strains exhibiting mexAB-oprM overtranscription; Kruskal-Wallis equality of population rank test, p>0.05.
(TIF)

S1 Table Accession numbers (id) of the *Pseudomonas aeruginosa* strains used in this work. (DOCX)

S2 Table Genetic variations identified in the *mexR*, *nalC* and *nalD* repressor genes in P. aeruginosa strains. (DOCX)

S3 Table Features and GenBank accession numbers of *Pseudomonas aeruginosa* ST233 complete genomes downloaded for this study. (DOCX)

S1 Dataset *mexA* transcript expression. (XLSX)

## References

[1] Sousa AM, Pereira MO. *Pseudomonas aeruginosa* Diversification during Infection Development in Cystic Fibrosis Lungs-A Review. Pathogens. 2014 Aug 18;3(3):680–703. doi: 10.3390/pathogens3030680 25438018 PMC4243435

[2] Aboushleib HM, Omar HM, Abozahra R, Elsheredy A, Baraka K. Correlation of quorum sensing and virulence factors in *Pseudomonas aeruginosa* isolates in Egypt. J Infect Dev Ctries. 2015 Oct 29;9(10):1091–9. doi: 10.3855/jidc.6492 26517484

[3] Maatallah M, Cheriaa J, Backhrouf A, Iversen A, Grundmann H, Do T, et al. Population structure of *Pseudomonas aeruginosa* from five Mediterranean countries: evidence for frequent recombination and epidemic occurrence of CC235. PLoS One. 2011;6(10):e25617. doi: 10.1371/journal.pone.0025617 Epub 2011 Oct 3. 21984923 PMC3184967

[4] World Health Organization. Global priority list of antibiotic-resistant bacteria. 2021. Available from: https://www.doherty.edu.au/news-events/news/who-global-priority-pathogens-list-of-antibiotic-resistant-bacteria.

[5] Oliver A, Mulet X, López-Causapé C, Juan C. The increasing threat of *Pseudomonas aeruginosa* high-risk clones. Drug Resist Updat. 2015 Jul-Aug;21–22:41–59. doi: 10.1016/j.drup.2015.08.002 Epub 2015 Aug 10. 26304792

[6] Aguilar-Rodea P, Zúñiga G, Rodríguez-Espino BA, Olivares Cervantes AL, Gamiño Arroyo AE, Moreno-Espinosa S, et al. Identification of extensive drug resistant *Pseudomonas aeruginosa* strains: New clone ST1725 and high-risk clone ST233. PLoS One. 2017 Mar 2;12(3):e0172882. doi: 10.1371/journal.pone.0172882 28253282 PMC5333833

[7] García-Castillo M, Del Campo R, Morosini MI, Riera E, Cabot G, Willems R, et al. Wide dispersion of ST175 clone despite high genetic diversity of carbapenem-nonsusceptible *Pseudomonas aeruginosa* clinical strains in 16 Spanish hospitals. J Clin Microbiol. 2011 Aug;49(8):2905–10. doi: 10.1128/JCM.00753-11 Epub 2011 Jun 22. 21697331 PMC3147750

[8] Correa A, Del Campo R, Perenguez M, Blanco VM, Rodríguez-Baños M, Perez F, et al. Dissemination of high-risk clones of extensively drug-resistant *Pseudomonas aeruginosa* in colombia. Antimicrob Agents Chemother. 2015 Apr;59(4):2421–5. doi: 10.1128/AAC.03926-14 Epub 2015 Jan 20. 25605362 PMC4356786

[9] Pragasam AK, Veeraraghavan B, Anandan S, Narasiman V, Sistla S, Kapil A, et al. Dominance of international high-risk clones in carbapenemase-producing *Pseudomonas aeruginosa*: Multicentric molecular epidemiology report from India. Indian J Med Microbiol. 2018 Jul-Sep;36(3):344–351. doi: 10.4103/ijmm.IJMM_18_294 30429385

[10] Lister PD, Wolter DJ, Hanson ND. Antibacterial-resistant *Pseudomonas aeruginosa*: clinical impact and complex regulation of chromosomally encoded resistance mechanisms. Clin Microbiol Rev. 2009 Oct;22(4):582–610. doi: 10.1128/CMR.00040-09 19822890 PMC2772362

[11] Poole K. *Pseudomonas aeruginosa*: resistance to the max. Front Microbiol. 2011 Apr 5;2:65. doi: 10.3389/fmicb.2011.00065 21747788 PMC3128976

[12] Masuda N, Sakagawa E, Ohya S, Gotoh N, Tsujimoto H, Nishino T. Substrate specificities of MexAB-OprM, MexCD-OprJ, and MexXY-oprM efflux pumps in *Pseudomonas aeruginosa*. Antimicrob Agents Chemother. 2000 Dec;44(12):3322–7. doi: 10.1128/AAC.44.12.3322-3327.2000 11083635 PMC90200

[13] De Kievit TR, Parkins MD, Gillis RJ, Srikumar R, Ceri H, Poole K, et al. Multidrug efflux pumps: expression patterns and contribution to antibiotic resistance in *Pseudomonas aeruginosa* biofilms. Antimicrob Agents Chemother. 2001 Jun;45(6):1761–70. doi: 10.1128/AAC.45.6.1761-1770.2001 11353623 PMC90543

[14] Hirakata Y, Srikumar R, Poole K, Gotoh N, Suematsu T, Kohno S, et al. Multidrug efflux systems play an important role in the invasiveness of *Pseudomonas aeruginosa*. J Exp Med. 2002 Jul 1;196(1):109–18. doi: 10.1084/jem.20020005 12093875 PMC2194012

[15] Nehme D, Li XZ, Elliot R, Poole K. Assembly of the MexAB-OprM multidrug efflux system of *Pseudomonas aeruginosa*: identification and characterization of mutations in mexA compromising MexA multimerization and interaction with MexB. J Bacteriol. 2004 May;186(10):2973–83. doi: 10.1128/JB.186.10.2973-2983.2004 15126457 PMC400598

[16] Sobel ML, Hocquet D, Cao L, Plesiat P, Poole K. Mutations in PA3574 (nalD) lead to increased MexAB-OprM expression and multidrug resistance in laboratory and clinical isolates of *Pseudomonas aeruginosa*. Antimicrob Agents Chemother. 2005 May;49(5):1782–6. doi: 10.1128/AAC.49.5.1782-1786.2005 15855496 PMC1087681

[17] Pan YP, Xu YH, Wang ZX, Fang YP, Shen JL. Overexpression of MexAB-OprM efflux pump in carbapenem-resistant *Pseudomonas aeruginosa*. Arch Microbiol. 2016 Aug;198(6):565–71. doi: 10.1007/s00203-016-1215-7 Epub 2016 Apr 8. 27060003

[18] Kanagaratnam R, Sheikh R, Alharbi F, Kwon DH. An efflux pump (MexAB-OprM) of *Pseudomonas aeruginosa* is associated with antibacterial activity of Epigallocatechin-3-gallate (EGCG). Phytomedicine. 2017 Dec 1;36:194–200. doi: 10.1016/j.phymed.2017.10.010 Epub 2017 Oct 12. 29157815

[19] Goli HR, Nahaei MR, Rezaee MA, Hasani A, Kafil HS, Aghazadeh M, et al. Role of MexAB-OprM and MexXY-OprM efflux pumps and class 1 integrons in resistance to antibiotics in burn and Intensive Care Unit isolates of *Pseudomonas aeruginosa*. J Infect Public Health. 2018 May-Jun;11(3):364–372. doi: 10.1016/j.jiph.2017.09.016 Epub 2017 Oct 6. 28993173

[20] Poole K. Efflux-mediated multiresistance in Gram-negative bacteria. Clin Microbiol Infect. 2004 Jan;10(1):12–26. doi: 10.1111/j.1469-0691.2004.00763.x 14706082

[21] Huffman JL, Brennan RG. Prokaryotic transcription regulators: more than just the helix-turn-helix motif. Curr Opin Struct Biol. 2002 Feb;12(1):98–106. doi: 10.1016/s0959-440x(02)00295-6 11839496

[22] Poole K, Gotoh N, Tsujimoto H, Zhao Q, Wada A, Yamasaki T, et al. Overexpression of the mexC-mexD-oprJ efflux operon in nfxB-type multidrug-resistant strains of *Pseudomonas aeruginosa*. Mol Microbiol. 1996 Aug;21(4):713–24. doi: 10.1046/j.1365-2958.1996.281397.x 8878035

[23] Morita Y, Cao L, Gould VC, Avison MB, Poole K. nalD encodes a second repressor of the mexAB-oprM multidrug efflux operon of *Pseudomonas aeruginosa*. J Bacteriol. 2006 Dec;188(24):8649–54. doi: 10.1128/JB.01342-06 Epub 2006 Oct 6. 17028276 PMC1698243

[24] Suresh M, Nithya N, Jayasree PR, Vimal KP, Manish Kumar PR. Mutational analyses of regulatory genes, mexR, nalC, nalD and mexZ of mexAB-oprM and mexXY operons, in efflux pump hyperexpressing multidrug-resistant clinical isolates of *Pseudomonas aeruginosa*. World J Microbiol Biotechnol. 2018 May 30;34(6):83. doi: 10.1007/s11274-018-2465-0 29846800

[25] Guénard S, Muller C, Monlezun L, Benas P, Broutin I, Jeannot K, et al. Multiple mutations lead to MexXY-OprM-dependent aminoglycoside resistance in clinical strains of *Pseudomonas aeruginosa*. Antimicrob Agents Chemother. 2014;58(1):221–8. doi: 10.1128/AAC.01252-13 Epub 2013 Oct 21. Erratum in: Antimicrob Agents Chemother. 2014 Mar;58(3):1833. 24145539 PMC3910787

[26] Kos VN, Déraspe M, McLaughlin RE, Whiteaker JD, Roy PH, Alm RA, et al. The resistome of *Pseudomonas aeruginosa* in relationship to phenotypic susceptibility. Antimicrob Agents Chemother. 2015 Jan;59(1):427–36. doi: 10.1128/AAC.03954-14 Epub 2014 Nov 3. . 25367914 PMC4291382

[27] Horna G, López M, Guerra H, Saénz Y, Ruiz J. Interplay between MexAB-OprM and MexEF-OprN in clinical isolates of *Pseudomonas aeruginosa*. Sci Rep. 2018 Nov 7;8(1):16463. doi: 10.1038/s41598-018-34694-z 30405166 PMC6220265

[28] Sanz-García F, Hernando-Amado S, Martínez JL. Mutational Evolution of *Pseudomonas aeruginosa* Resistance to Ribosome-Targeting Antibiotics. Front Genet. 2018 Oct 18;9:451. doi: 10.3389/fgene.2018.00451 30405685 PMC6200844

[29] Ziha-Zarifi I, Llanes C, Köhler T, Pechere JC, Plesiat P. In vivo emergence of multidrug-resistant mutants of *Pseudomonas aeruginosa* overexpressing the active efflux system MexA-MexB-OprM. Antimicrob Agents Chemother. 1999 Feb;43(2):287–91. doi: 10.1128/AAC.43.2.287 9925520 PMC89065

[30] Poole K. Stress responses as determinants of antimicrobial resistance in *Pseudomonas aeruginosa*: multidrug efflux and more. Can J Microbiol. 2014 Dec;60(12):783–91. doi: 10.1139/cjm-2014-0666 25388098

[31] Magiorakos AP, Srinivasan A, Carey RB, Carmeli Y, Falagas ME, Giske CG, et al. Multidrug-resistant, extensively drug-resistant and pandrug-resistant bacteria: an international expert proposal for interim standard definitions for acquired resistance. Clin Microbiol Infect. 2012 Mar;18(3):268–81. doi: 10.1111/j.1469-0691.2011.03570.x Epub 2011 Jul 27. 21793988

[32] The Clinical and Laboratory Standards Institute. M100S Performance Standards for Antimicrobial Susceptibility Testing. Clinical and Laboratory Standards Institute, Wayne, PA. 2020.

[33] Smith EC, Brigman HV, Anderson JC, Emery CL, Bias TE, Bergen PJ, et al. Performance of Four Fosfomycin Susceptibility Testing Methods against an International Collection of Clinical Pseudomonas aeruginosa Isolates. J Clin Microbiol. 2020 Sep 22;58(10):e01121–20. doi: 10.1128/JCM.01121-20 32669380 PMC7512173

[34] Dortet L, Poirel L, Nordmann P. Rapid detection of carbapenemase-producing *Pseudomonas* spp. J Clin Microbiol. 2012 Nov;50(11):3773–6. doi: 10.1128/JCM.01597-12 Epub 2012 Sep 12. 22972829 PMC3486216

[35] Mesaros N, Glupczynski Y, Avrain L, Caceres NE, Tulkens PM, Van Bambeke F. A combined phenotypic and genotypic method for the detection of Mex efflux pumps in *Pseudomonas aeruginosa*. J Antimicrob Chemother. 2007 Mar;59(3):378–86. doi: 10.1093/jac/dkl504 Epub 2007 Feb 8. 17289770

[36] Lutgring JD, Machado MJ, Benahmed FH, Conville P, Shawar RM, Patel J, et al. FDA-CDC Antimicrobial Resistance Isolate Bank: a Publicly Available Resource To Support Research, Development, and Regulatory Requirements. J Clin Microbiol. 2018 Jan 24;56(2):e01415–17. doi: 10.1128/JCM.01415-17 29118174 PMC5786719

[37] Rampioni G, Pillai CR, Longo F, Bondì R, Baldelli V, Messina M, et al. Effect of efflux pump inhibition on *Pseudomonas aeruginosa* transcriptome and virulence. Sci Rep. 2017 Sep 12;7(1):11392. doi: 10.1038/s41598-017-11892-9 28900249 PMC5596013

[38] Untergasser A, Cutcutache I, Koressaar T, Ye J, Faircloth BC, Remm M, et al. Primer3—new capabilities and interfaces. Nucleic Acids Res. 2012 Aug;40(15):e115. doi: 10.1093/nar/gks596 Epub 2012 Jun 22. 22730293 PMC3424584

[39] De Alba Aguayo D, Rueda A. Problema bioquímico: Determinación del ciclo umbral y la eficiencia para la PCR cuantitativa en tiempo real. *Revista de educación bioquímica*, 2013;32(1), 36–39.

[40] Curran B, Jonas D, Grundmann H, Pitt T, Dowson CG. Development of a multilocus sequence typing scheme for the opportunistic pathogen *Pseudomonas aeruginosa*. J Clin Microbiol. 2004 Dec;42(12):5644–9. doi: 10.1128/JCM.42.12.5644-5649.2004 15583294 PMC535286

[41] Jolley KA, Bray JE, Maiden MCJ. Open-access bacterial population genomics: BIGSdb software, the PubMLST.org website and their applications. Wellcome Open Res. 2018 Sep 24;3:124. doi: 10.12688/wellcomeopenres.14826.1 30345391 PMC6192448

[42] Larkin MA, Blackshields G, Brown NP, Chenna R, McGettigan PA, McWilliam H, et al. Clustal W and Clustal X version 2.0. Bioinformatics. 2007 Nov 1;23(21):2947–8. doi: 10.1093/bioinformatics/btm404 Epub 2007 Sep 10. 17846036

[43] Gouy M, Guindon S, Gascuel O. SeaView version 4: A multiplatform graphical user interface for sequence alignment and phylogenetic tree building. Mol Biol Evol. 2010 Feb;27(2):221–4. doi: 10.1093/molbev/msp259 Epub 2009 Oct 23. 19854763

[44] Geospiza, Inc. FinchTV ver. 1.4.0. A brilliant trace viewer. 2006. Available from http://www.geospiza.com/Products/finchtv.shtml.

[45] Rozas J, Ferrer-Mata A, Sánchez-DelBarrio JC, Guirao-Rico S, Librado P, Ramos-Onsins SE, et al. DnaSP 6: DNA Sequence Polymorphism Analysis of Large Data Sets. Mol Biol Evol. 2017 Dec 1;34(12):3299–3302. doi: 10.1093/molbev/msx248 29029172

[46] Zhou Z, Alikhan NF, Sergeant MJ, Luhmann N, Vaz C, Francisco AP, et al. GrapeTree: visualization of core genomic relationships among 100,000 bacterial pathogens. Genome Res. 2018 Sep;28(9):1395–1404. doi: 10.1101/gr.232397.117 Epub 2018 Jul 26. 30049790 PMC6120633

[47] Gonçalves B, Carriço JA, Francisco AP, Vaz C, Ramírez M. PhyloViZ: phylogenetic tree vizualisation—*Pseudomonas aeruginosa* isolates. PubMLST *Pseudomonas aeruginosa*. 2018. Available from: https://online.phyloviz.net/

[48] Huson DH, Bryant D. Application of phylogenetic networks in evolutionary studies. Mol Biol Evol. 2006 Feb;23(2):254–67. doi: 10.1093/molbev/msj030 Epub 2005 Oct 12. 16221896

[49] StataCorp. STATA/MP 14.1. Stata Statistical Software: Release 13. College Station, TX: StataCorp, L.P. 2013.

[50] RStudio Team. RStudio: Integrated Development for R. RStudio, Inc., Boston, M.A. 2015.

[51] de Almeida Silva KCF, Calomino MA, Deutsch G, de Castilho SR, de Paula GR, Esper LMR, et al. Molecular characterization of multidrug-resistant (MDR) *Pseudomonas aeruginosa* isolated in a burn center. Burns. 2017 Feb;43(1):137–143. doi: 10.1016/j.burns.2016.07.002 Epub 2016 Aug 29. 27595453

[52] Sabuda DM, Laupland K, Pitout J, Dalton B, Rabin H, Louie T, et al. Utilization of colistin for treatment of multidrug-resistant *Pseudomonas aeruginosa*. Can J Infect Dis Med Microbiol. 2008 Nov;19(6):413–8. doi: 10.1155/2008/743197 19436571 PMC2663472

[53] Dößelmann B, Willmann M, Steglich M, Bunk B, Nübel U, Peter S, et al. Rapid and Consistent Evolution of Colistin Resistance in Extensively Drug-Resistant *Pseudomonas aeruginosa* during Morbidostat Culture. Antimicrob Agents Chemother. 2017 Aug 24;61(9):e00043–17. doi: 10.1128/AAC.00043-17 28630206 PMC5571341

[54] Pappa O, Beloukas A, Vantarakis A, Mavridou A, Kefala AM, Galanis A. Molecular Characterization and Phylogenetic Analysis of *Pseudomonas aeruginosa* Isolates Recovered from Greek Aquatic Habitats Implementing the Double-Locus Sequence Typing Scheme. Microb Ecol. 2017 Jul;74(1):78–88. doi: 10.1007/s00248-016-0920-8 Epub 2016 Dec 28. 28032128

[55] Zhang Y, Gu AZ, Cen T, Li X, He M, Li D, et al. Sub-inhibitory concentrations of heavy metals facilitate the horizontal transfer of plasmid-mediated antibiotic resistance genes in water environment. Environ Pollut. 2018 Jun;237:74–82. doi: 10.1016/j.envpol.2018.01.032 Epub 2018 Feb 21. 29477117

[56] Bazzi W, Abou Fayad AG, Nasser A, Haraoui LP, Dewachi O, Abou-Sitta G, et al. Heavy Metal Toxicity in Armed Conflicts Potentiates AMR in *A*. *baumannii* by Selecting for Antibiotic and Heavy Metal Co-resistance Mechanisms. Front Microbiol. 2020 Feb 3;11:68. doi: 10.3389/fmicb.2020.00068 32117111 PMC7008767

[57] Yang K, Zhang Y. Reversal of heavy metal-induced antibiotic resistance by dandelion root extracts and taraxasterol. J Med Microbiol. 2020 Aug;69(8):1049–1061. doi: 10.1099/jmm.0.001226 32602832

[58] Zhong Q, Cruz-Paredes C, Zhang S, Rousk J. Can heavy metal pollution induce bacterial resistance to heavy metals and antibiotics in soils from an ancient land-mine? J Hazard Mater. 2021 Jun 5;411:124962. doi: 10.1016/j.jhazmat.2020.124962 Epub 2021 Jan 4. 33440279

[59] Mudau M, Jacobson R, Minenza N, Kuonza L, Morris V, Engelbrecht H, et al. Outbreak of multi-drug resistant *Pseudomonas aeruginosa* bloodstream infection in the haematology unit of a South African Academic Hospital. PLoS One. 2013;8(3):e55985. doi: 10.1371/journal.pone.0055985 Epub 2013 Mar 14. 23516393 PMC3597724

[60] Perez F, Hujer AM, Marshall SH, Ray AJ, Rather PN, Suwantarat N, et al. Extensively drug-resistant *Pseudomonas aeruginosa* isolates containing blaVIM-2 and elements of Salmonella genomic island 2: a new genetic resistance determinant in Northeast Ohio. Antimicrob Agents Chemother. 2014 Oct;58(10):5929–35. doi: 10.1128/AAC.02372-14 Epub 2014 Jul 28. 25070102 PMC4187935

[61] Samuelsen O, Toleman MA, Sundsfjord A, Rydberg J, Leegaard TM, Walder M, et al. Molecular epidemiology of metallo-beta-lactamase-producing *Pseudomonas aeruginosa* isolates from Norway and Sweden shows import of international clones and local clonal expansion. Antimicrob Agents Chemother. 2010 Jan;54(1):346–52. doi: 10.1128/AAC.00824-09 Epub 2009 Nov 2. 19884381 PMC2798561

[62] Tsutsui A, Suzuki S, Yamane K, Matsui M, Konda T, Marui E, et al. Genotypes and infection sites in an outbreak of multidrug-resistant Pseudomonas aeruginosa. J Hosp Infect. 2011 Aug;78(4):317–22. doi: 10.1016/j.jhin.2011.04.013 21689862

[63] Zafer MM, Al-Agamy MH, El-Mahallawy HA, Amin MA, El Din Ashour S. Dissemination of VIM-2 producing Pseudomonas aeruginosa ST233 at tertiary care hospitals in Egypt. BMC Infect Dis. 2015 Mar 12;15:122. doi: 10.1186/s12879-015-0861-8 25880997 PMC4396152

[64] Freschi L, Bertelli C, Jeukens J, Moore MP, Kukavica-Ibrulj I, Emond-Rheault JG, et al. Genomic characterisation of an international *Pseudomonas aeruginosa* reference panel indicates that the two major groups draw upon distinct mobile gene pools. FEMS Microbiol Lett. 2018 Jul 1;365(14). doi: 10.1093/femsle/fny120 29897457

[65] Cholley P, Thouverez M, Hocquet D, van der Mee-Marquet N, Talon D, Bertrand X. Most multidrug-resistant *Pseudomonas aeruginosa* isolates from hospitals in eastern France belong to a few clonal types. J Clin Microbiol. 2011 Jul;49(7):2578–83. doi: 10.1128/JCM.00102-11 Epub 2011 May 18. 21593258 PMC3147838

[66] Guzvinec M, Izdebski R, Butic I, Jelic M, Abram M, Koscak I, et al. Sequence types 235, 111, and 132 predominate among multidrug-resistant *Pseudomonas aeruginosa* clinical isolates in Croatia. Antimicrob Agents Chemother. 2014 Oct;58(10):6277–83. doi: 10.1128/AAC.03116-14 Epub 2014 Jul 28. 25070098 PMC4187922

[67] Pirnay JP, Bilocq F, Pot B, Cornelis P, Zizi M, Van Eldere J, et al. *Pseudomonas aeruginosa* population structure revisited. PLoS One. 2009 Nov 13;4(11):e7740. doi: 10.1371/journal.pone.0007740 19936230 PMC2777410

[68] Arabestani MR, Rajabpour M, Yousefi Mashouf R, Alikhani MY, Mousavi SM. Expression of efflux pump MexAB-OprM and OprD of *Pseudomonas aeruginosa* strains isolated from clinical samples using qRT-PCR. Arch Iran Med. 2015 Feb;18(2):102–8. doi: 015182/AIM.008 25644798

[69] Ozer B, Duran N, Onlen Y, Savas L. Efflux pump genes and antimicrobial resistance of *Pseudomonas aeruginosa* strains isolated from lower respiratory tract infections acquired in an intensive care unit. J Antibiot (Tokyo). 2012 Jan;65(1):9–13. doi: 10.1038/ja.2011.102 Epub 2011 Nov 16. 22086166

[70] Vedel G. Simple method to determine beta-lactam resistance phenotypes in *Pseudomonas aeruginosa* using the disc agar diffusion test. J Antimicrob Chemother. 2005 Oct;56(4):657–64. doi: 10.1093/jac/dki303 Epub 2005 Sep 6. 16144872

[71] Yoneda K, Chikumi H, Murata T, Gotoh N, Yamamoto H, Fujiwara H, et al. Measurement of *Pseudomonas aeruginosa* multidrug efflux pumps by quantitative real-time polymerase chain reaction. FEMS Microbiol Lett. 2005 Feb 1;243(1):125–31. doi: 10.1016/j.femsle.2004.11.048 15668010

[72] Poonsuk K, Tribuddharat C, Chuanchuen R. Simultaneous overexpression of multidrug efflux pumps in *Pseudomonas aeruginosa* non-cystic fibrosis clinical isolates. Can J Microbiol. 2014 Jul;60(7):437–43. doi: 10.1139/cjm-2014-0239 Epub 2014 May 12. 24909060

[73] Vargas-Blanco DA, Shell SS. Regulation of mRNA Stability During Bacterial Stress Responses. Front Microbiol. 2020 Sep 9;11:2111. doi: 10.3389/fmicb.2020.02111 33013770 PMC7509114

[74] Chen W, Wang D, Zhou W, Sang H, Liu X, Ge Z, et al. Novobiocin binding to NalD induces the expression of the MexAB-OprM pump in *Pseudomonas aeruginosa*. Mol Microbiol. 2016 Jun;100(5):749–58. doi: 10.1111/mmi.13346 Epub 2016 Mar 16. 26844397

[75] Quale J, Bratu S, Gupta J, Landman D. Interplay of efflux system, ampC, and oprD expression in carbapenem resistance of *Pseudomonas aeruginosa* clinical isolates. Antimicrob Agents Chemother. 2006 May;50(5):1633–41. doi: 10.1128/AAC.50.5.1633-1641.2006 16641429 PMC1472219

[76] Heacock-Kang Y, Sun Z, Zarzycki-Siek J, Poonsuk K, McMillan IA, Chuanchuen R, et al. Two Regulators, PA3898 and PA2100, Modulate the *Pseudomonas aeruginosa* Multidrug Resistance MexAB-OprM and EmrAB Efflux Pumps and Biofilm Formation. Antimicrob Agents Chemother. 2018 Nov 26;62(12):e01459–18. doi: 10.1128/AAC.01459-18 30297364 PMC6256797

[77] Taiaroa G, Samuelsen Ø, Kristensen T, Økstad OAL, Heikal A. Complete Genome Sequence of *Pseudomonas aeruginosa* K34-7, a Carbapenem-Resistant Isolate of the High-Risk Sequence Type 233. Microbiol Resour Announc. 2018 Aug 2;7(4):e00886–18. doi: 10.1128/MRA.00886-18 30533874 PMC6256419

