# Supplementary figures and images for: Nucleotide substitutions in the mexR, nalC and nalD regulator genes of the MexAB-OprM efflux pump are maintained in Pseudomonas aeruginosa genetic lineages
Source: PLoS One. 2022 May 10;17(5):e0266742. doi: 10.1371/journal.pone.0266742 (PMC9089866; doi:10.1371/journal.pone.0266742)

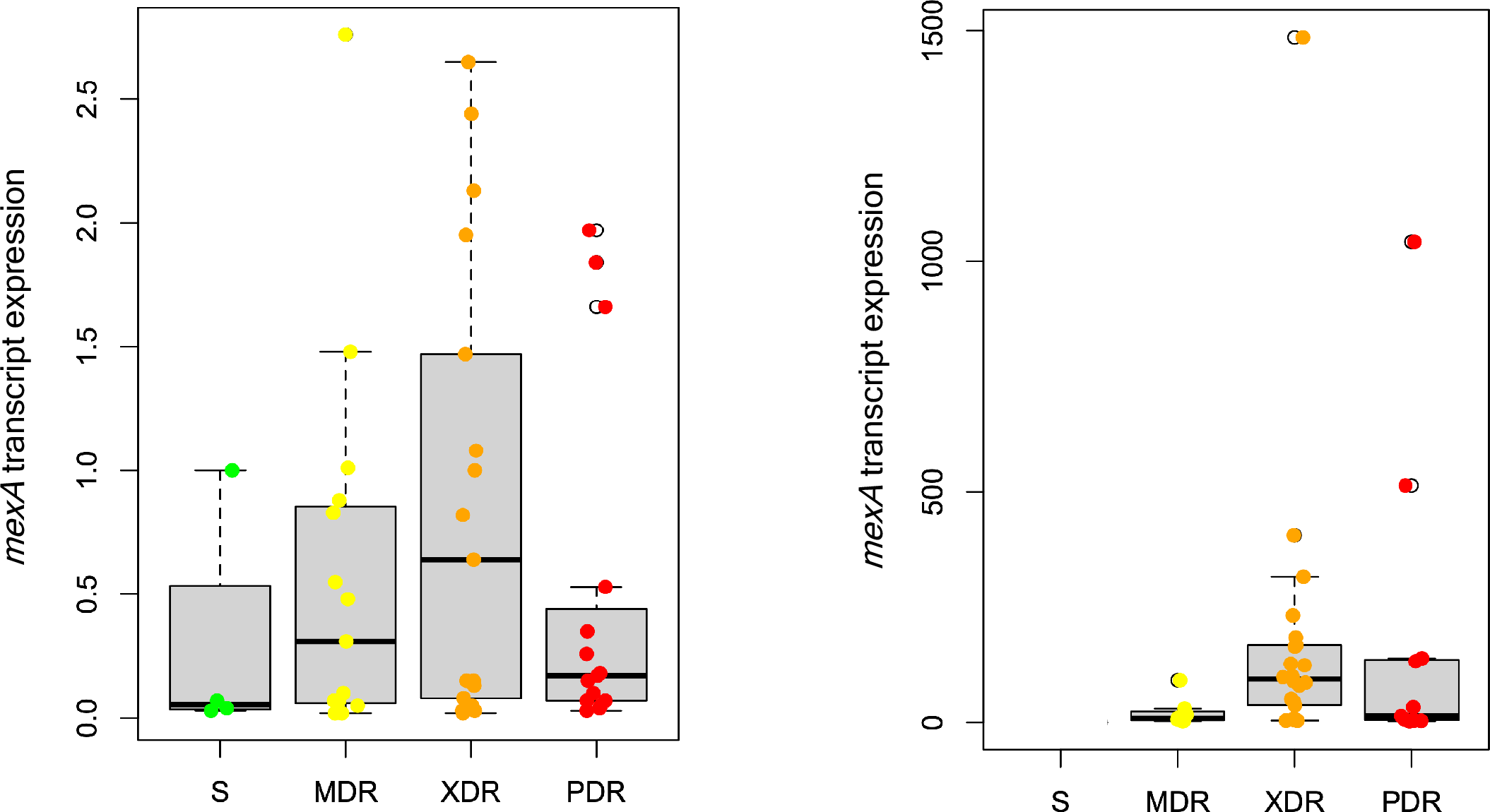

Supplement: S1 Fig — Transcript expression Ratio between the target gene mexA and the reference gene rpsL. A: Strains exhibiting mexAB-oprM basal level; B: Strains exhibiting mexAB-oprM overtranscription; Kruskal-Wallis equality of population rank test, p>0.05. (TIF) [file pone.0266742.s001.tif]
