# Supplementary material for: Nucleotide substitutions in the mexR, nalC and nalD regulator genes of the MexAB-OprM efflux pump are maintained in Pseudomonas aeruginosa genetic lineages
Source: PLoS One. 2022 May 10;17(5):e0266742. doi: 10.1371/journal.pone.0266742 (PMC9089866; doi:10.1371/journal.pone.0266742)
Supplement: S1 Table — (DOCX) [file pone.0266742.s002.docx]

**S1 Table**

| **Id at** [**PubMLST.org**](http://PubMLST.org) | **Isolate** | **ST** | **Id assigned in this manuscript** | **ST Source** |
| --- | --- | --- | --- | --- |
| **6071** | **HIM111** | **111** | **12A** | **ST111: Samuelsen *et al.*, 2010; Cholley *et al.*, 2011; García-Castillo *et al.*, 2011; Maatallah *et al.,* 2011; Guzvinec *et al.,* 2014; Correa *et al.,* 2015; Oliver *et al.*, 2015; Freschi *et al.,* 2018; In this work.** |
| **6052** | **HIM3/26** | **1725** | **18H** | **Aguilar-Rodea *et al.*, 2017** |
| **6042** | **HIM3/16** | **1725** | **54H** | **Aguilar-Rodea *et al.*, 2017** |
| **2170** | **HIM3** | **1725** | **3H** | **Aguilar-Rodea *et al.*, 2017** |
| **6037** | **HIM3/11** | **1725** | **14H** | **Aguilar-Rodea *et al.*, 2017** |
| **6038** | **HIM3/12** | **1725** | **15H** | **Aguilar-Rodea *et al.*, 2017** |
| **6048** | **HIM3/22** | **1725** | **39H** | **Aguilar-Rodea *et al.*, 2017** |
| **6054** | **HIM3/28** | **1725** | **55H** | **Aguilar-Rodea *et al.*, 2017** |
| **6031** | **HIM3/5** | **1725** | **8H** | **Aguilar-Rodea *et al.*, 2017** |
| **6059** | **HIM3/33** | **1725** | **53H** | **Aguilar-Rodea *et al.*, 2017** |
| **6049** | **HIM3/23** | **1725** | **38H** | **Aguilar-Rodea *et al.*, 2017** |
| **6043** | **HIM3/17** | **1725** | **43H** | **Aguilar-Rodea *et al.*, 2017** |
| **6028** | **HIM3/2** | **1725** | **5H** | **Aguilar-Rodea *et al.*, 2017** |
| **6040** | **HIM3/14** | **1725** | **25H** | **Aguilar-Rodea *et al.*, 2017** |
| **6029** | **HIM3/3** | **1725** | **6H** | **Aguilar-Rodea *et al.*, 2017** |
| **6030** | **HIM3/4** | **1725** | **7H** | **Aguilar-Rodea *et al.*, 2017** |
| **6034** | **HIM3/8** | **1725** | **30H** | **Aguilar-Rodea *et al.*, 2017** |
| **6036** | **HIM3/10** | **1725** | **31H** | **Aguilar-Rodea *et al.*, 2017** |
| **6045** | **HIM3/19** | **1725** | **37H** | **Aguilar-Rodea *et al.*, 2017** |
| **6032** | **HIM3/6** | **1725** | **11H** | **Aguilar-Rodea *et al.*, 2017** |
| **6033** | **HIM3/7** | **1725** | **12H** | **Aguilar-Rodea *et al.*, 2017** |
| **6041** | **HIM3/15** | **1725** | **26H** | **Aguilar-Rodea *et al.*, 2017** |
| **6044** | **HIM3/18** | **1725** | **36H** | **Aguilar-Rodea *et al.*, 2017** |
| **6047** | **HIM3/21** | **1725** | **40H** | **Aguilar-Rodea *et al.*, 2017** |
| **6050** | **HIM3/24** | **1725** | **41H** | **Aguilar-Rodea *et al.*, 2017** |
| **6056** | **HIM3/30** | **1725** | **50H** | **Aguilar-Rodea *et al.*, 2017** |
| **6035** | **HIM3/9** | **1725** | **33H** | **Aguilar-Rodea *et al.*, 2017** |
| **6053** | **HIM3/27** | **1725** | **48H** | **Aguilar-Rodea *et al.*, 2017** |
| **6027** | **HIM3/1** | **1725** | **4H** | **Aguilar-Rodea *et al.*, 2017** |
| **6039** | **HIM3/13** | **1725** | **16H** | **Aguilar-Rodea *et al.*, 2017** |
| **6051** | **HIM3/25** | **1725** | **42H** | **Aguilar-Rodea *et al.*, 2017** |
| **6055** | **HIM3/29** | **1725** | **49H** | **Aguilar-Rodea *et al.*, 2017** |
| **6046** | **HIM3/20** | **1725** | **35H** | **Aguilar-Rodea *et al.*, 2017** |
| **6057** | **HIM3/31** | **1725** | **51H** | **Aguilar-Rodea *et al.*, 2017** |
| **3207** | **HIM18** | **2244** | **46H** | **Aguilar-Rodea *et al.*, 2017** |
| **3208** | **HIM19** | **2245** | **47H** | **Aguilar-Rodea *et al.*, 2017** |
| **3210** | **HIM21** | **2247*** | **52H** | **In this work** |
| **3206** | **HIM17** | **2243** | **45H** | **Aguilar-Rodea *et al.*, 2017** |
| **2150** | **HIM1** | **1723** | **1H** | **Aguilar-Rodea *et al.*, 2017** |
| **2156** | **HIM8** | **1730** | **32H** | **Aguilar-Rodea *et al.*, 2017** |
| **6058** | **HIM3/32** | **1725** | **56H** | **Aguilar-Rodea *et al.*, 2017** |
| **3209** | **HIM20** | **2246** | **44H** | **Aguilar-Rodea *et al.*, 2017** |
| **5901** | **HIM35** | **2566*** | **10A** | **In this work** |
| **2152** | **HIM4** | **1726** | **10H** | **Aguilar-Rodea *et al.*, 2017** |
| **2153** | **HIM5** | **1727** | **27H** | **Aguilar-Rodea *et al.*, 2017** |
| **2151** | **HIM2** | **1724** | **9H** | **Aguilar-Rodea *et al.*, 2017** |
| **6026** | **HIM2/1** | **1724** | **2H** | **Aguilar-Rodea *et al.*, 2017** |
| **2154** | **HIM6** | **1728** | **28H** | **Aguilar-Rodea *et al.*, 2017** |
| **2159** | **HIM11** | **1733** | **17H** | **Aguilar-Rodea *et al.*, 2017** |
| **5902** | **HIM36** | **2567*** | **11A** | **In this work** |
| **6020** | **HIM40** | **2710*** | **68H** | **In this work** |
| **6022** | **HIM42** | **2716*** | **70H** | **In this work** |
| **6018** | **HIM38** | **2704*** | **63H** | **In this work** |
| **6063** | **HIM40/1** | **2710*** | **67H** | **In this work** |
| **6023** | **HIM43** | **2731*** | **72H** | **In this work** |
| **6021** | **HIM41** | **2713*** | **69H** | **In this work** |
| **6024** | **HIM44** | **2732*** | **73H** | **In this work** |
| **3212** | **HIM23** | **2249*** | **2A** | **In this work** |
| **5900** | **HIM34** | **2565*** | **9A** | **In this work** |
| **6062** | **HIM28/1** | **2559*** | **71H** | **In this work** |
| **6066** | **HIM233/2** | **233** | **65H** | **ST233: Zafer et al., 2015; Pragasam et al., 2018; Mudau et al., 2013, In this work** |
| **6067** | **HIM233/3** | **233** | **75H** | **ST233: Zafer et al., 2015; Pragasam et al., 2018; Mudau et al., 2013, In this work** |
| **6068** | **HIM233/4** | **233** | **76H** | **ST233: Zafer et al., 2015; Pragasam et al., 2018; Mudau et al., 2013, In this work** |
| **5895** | **HIM29** | **2560*** | **74H** | **In this work** |
| **5894** | **HIM28** | **2559*** | **66H** | **In this work** |
| **6065** | **HIM233/1** | **233** | **57H** | **ST233: Zafer et al., 2015; Aguilar-Rodea *et al.*, 2017: Pragasam et al., 2018; Mudau et al., 2013.** |
| **6064** | **HIM233** | **233** | **59H** | **ST233: Zafer et al., 2015; Aguilar-Rodea *et al.*, 2017; Pragasam et al., 2018; Mudau et al., 2013.** |
| **6069** | **HIM233/5** | **233** | **77H** | **ST233: Zafer et al., 2015; Pragasam et al., 2018; Mudau et al., 2013, In this work** |
| **5896** | **HIM30** | **2561*** | **1A** | **In this work** |
| **2164** | **HIM16** | **1736** | **23H** | **Aguilar-Rodea *et al.*, 2017** |
| **6061** | **HIM16/1** | **1736** | **24H** | **Aguilar-Rodea *et al.*, 2017** |
| **5892** | **HIM26** | **2557*** | **61H** | **In this work** |
| **5903** | **HIM37** | **2568*** | **13A** | **In this work** |
| **6019** | **HIM39** | **2709*** | **64H** | **In this work** |
| **3211** | **HIM22** | **2248** | **60H** | **Aguilar-Rodea *et al.*, 2017** |
| **5893** | **HIM27** | **2558*** | **62H** | **In this work** |
| **6070** | **HIM112** | **112** | **58H** | **ST112: Aguilar-Rodea *et al.*, 2017; Chairat et al., 2019.** |
| **2162** | **HIM14** | **1735** | **22H** | **Aguilar-Rodea *et al.*, 2017** |
| **2163** | **HIM15** | **561** | **34H** | **Aguilar-Rodea *et al.*, 2017** |
| **2161** | **HIM13** | **1737** | **13H** | **Aguilar-Rodea *et al.*, 2017** |
| **2157** | **HIM9** | **1731** | **19H** | **Aguilar-Rodea *et al.*, 2017** |
| **5898** | **HIM32** | **2563*** | **4A** | **In this work** |
| **2160** | **HIM12** | **1734** | **21H** | **Aguilar-Rodea *et al.*, 2017** |
| **5897** | **HIM31** | **2562*** | **3A** | **In this work** |
| **3213** | **HIM24** | **2250*** | **7A** | **In this work** |
| **6072** | **HIM540** | **540** | **6A** | **ST540: Aguilar-Rodea *et al.*, 2017; Liu et al., 2018; Tada et al., 2019,** |
| **3214** | **HIM25** | **2251*** | **8A** | **In this work** |
| **5899** | **HIM33** | **2564*** | **5A** | **In this work** |
| **2155** | **HIM7** | **1729** | **29H** | **Aguilar-Rodea *et al.*, 2017** |
| **6060** | **HIM7/1** | **1729** | **14A** | **Aguilar-Rodea *et al.*, 2017** |
| **2158** | **HIM10** | **2226** | **20H** | **Aguilar-Rodea *et al.*, 2017** |

**Ids available at the public database for molecular typing: PubMLST.org.**

ST: sequence type; *: new ST identified in this work and deposited in the PubMLST *Pseudomonas aeruginosa* database; #H: nosocomial strains, #A: environmental strains.
