# Supplementary material for: Nucleotide substitutions in the mexR, nalC and nalD regulator genes of the MexAB-OprM efflux pump are maintained in Pseudomonas aeruginosa genetic lineages
Source: PLoS One. 2022 May 10;17(5):e0266742. doi: 10.1371/journal.pone.0266742 (PMC9089866; doi:10.1371/journal.pone.0266742)
Supplement: S2 Table — (DOCX) [file pone.0266742.s003.docx]

**S2 Table**

| **Repressor gene** | **Genetic**  **variation** | **H**  **n=77** | **A**  **n=14** | **Total n=91** | **Nucleotide Variations** | **Amino acid Variation** |
| --- | --- | --- | --- | --- | --- | --- |
| ***mexR*** | No substitution | 16 | 5 | 21 | - | - |
|  | Synonymous substitution  (n= 13) | 59 | 9 | 68 | ^15^G→C, ^18^T→C, ^33^C→T, ^60^G→A, ^96^A→G, ^168^C→T, ^201^G→A, ^264^C→T, ^327^G→A, ^378^G→T, ^384^G→A, ^411^G→A | **V_5_V, N_6_N, P_11_P,** V_20_V, T_22_T, **R_32_R**, D_56_D, **L_67_L**, **S_88_S, E_109_E**, V_126_V, **Q_128_Q**, Q_137_Q |
|  | Nonsynonymous substitution  (n= 3) | 57 | 8 | 65 | ^170^T→C, ^268^C→T, ^377^T→A | L_57_P, Q_90_*, **V_126_E** |
|  | Amino acid substitution | 55 | 8 | 63 | ^170^T→C, ^377^T→A | L_57_P, **V_126_E** |
|  | Stop | 2 | 0 | 2 | ^268^C→T | Q_90_* |
| ***nalC*** | No substitution | 0 | 1 | 1 | - | - |
|  | Synonymous substitution  (n= 19) | 61 | 9 | 70 | ^12^T→G, ^15^T→C, ^69^T→C, ^123^A→T, ^129^G→A, ^147^G→A, ^177^G→A, ^186^C→T, ^258^G→A, ^294^T→C, ^354^C→T, ^358^C→A, ^369^G→A, ^411^T→C, ^435^C→A, ^441^C→T, ^444^T→C, ^447^T→C, ^558^G→A | A_4_A, S_5_S, A_23_A, I_41_I, R_43_R, G_49_G, E_59_E, F_62_F, **T_86_T**, F_98_F, S_118_S, R_120_R, A_123_A, Y_137_Y, A_145_A, **V_147_V**, A_148_A, **P_149_P**, A_186_A |
|  | Nonsynonymous substitution  (n= 9) | 77 | 13 | 90 | ^△^105-116, ^130^G→A, ^212^G→A, ^237^T→A, ^434^C→T, ^457^G→C, ^459^G→T, ^556^G→A, ^625^A→C | (T_35_△, T_36_△, L_37_△, D_38_△, M_39_△), A_44_T, **G_71_E**, D_79_E, A_145_V, E_153_Q, E_153_D, A_186_T, **S_209_R** |
|  | Amino acid substitution | 77 | 13 | 90 | ^130^G→A, ^212^G→A, ^237^T→A, ^434^C→T, ^457^G→C, ^459^G→T, ^556^G→A, ^625^A→C | A_44_T, **G_71_E**, D_79_E, **A_145_V**, E_153_Q, E_153_D, A_186_T, **S_209_R** |
|  | Amino acid deletion | 1 | 0 | 1 | ^△^105-116 | T_35_△, T_36_△, L_37_△, D_38_△, M_39_△ |
| ***nalD*** | No substitution | 18 | 6 | 24 | - | - |
|  | Synonymous substitution  (n= 17) | 53 | 8 | 61 | ^78^G→A, ^120^C→T, ^135^C→A, ^153^C→T, ^165^C→T, ^169^C→T, ^231^C→A, ^276^C→T, ^295^T→C, ^297^G→A, ^303^G→T, ^333^C→T, ^450^T→C, ^477^G→A, ^504^G→A, ^540^C→T, ^555^T→C | **K_26_K**, A_40_A, G_45_G, F_51_F, A_55_A, L_57_L, S_77_S, C_92_C, L_99_L, **L_99_L**, T_101_T, I_111_I, R_150_R, P_159_P, A_168_A, D_180_D, **D_185_D** |
|  | Nonsynonymous subtitution  (n= 1) | 6 | 0 | 6 | ^136^G→A | A_46_T |
|  | Amino acid substitution | 6 | 0 | 6 | ^136^G→A | A_46_T |

H: nosocomial strains, A: environmental strains. Nucleotide variation: the number indicates the nucleotide position in the gene where the change occurs, the first letter indicates reference strain (*P. aeruginosa* PAO1) nucleotide, and the second letter indicates the nucleotide that substitutes the original. The symbol △nt means nucleotide deletion. Amino acid variation: A: alanine, C: cysteine, D: aspartic acid, E: glutamic acid, F: phenylalanine, G: glycine, H: histidine, I: isoleucine, K: lysine, L: leucine, M: methionine, N: asparagine, P: proline, Q: glutamine, R: Arginine, S: serine, T: threonine, V: valine, W: tryptophan, Y: tyrosine. The * means stop codon. First letter indicates reference strain (*P. aeruginosa* PAO1) amino acid, the number indicates the amino acid position where the change occurs, and the second letter indicates the amino acid that substitutes the original amino acid. In bold are SNPs previously reported by Suresh et al., 2018 and Quale et al., 2006 [24, 75].
