# Supplementary material for: Nucleotide substitutions in the mexR, nalC and nalD regulator genes of the MexAB-OprM efflux pump are maintained in Pseudomonas aeruginosa genetic lineages
Source: PLoS One. 2022 May 10;17(5):e0266742. doi: 10.1371/journal.pone.0266742 (PMC9089866; doi:10.1371/journal.pone.0266742)
Supplement: S3 Table — (DOCX) [file pone.0266742.s004.docx]

**S3 Table**

| **Strain** | **Number of**  **Genes** | **Sample Accession** | **Isolation**  **Source** | **Autor** | **Assembly Key**  **REFSEQ** | **Accession Number**  **REFSEQ** |
| --- | --- | --- | --- | --- | --- | --- |
| AR444 | 6412 | SAMN07291537 | missing | Benahmed *et al.,* 2018 | GCF_003073615.1 | NZ_CP029089.1 |
| AR_0230 | 6670 | SAMN04901620 | missing | Benahmed *et al.*, 2018 | GCF_002968695.1 | NZ_CP027174.1 |
| AR_0111 | 6708 | SAMN04014952 | missing | Conlan *et al.,* 2018 | GCF_003571805.1 | NZ_CP032257.1 |
| K34-7 | 6695 | SAMN08954392 | sputum | Taiaroa *et al.,* 2018 | GCF_003206535.1 | NZ_CP029707.1 |
| PA83 | 6782 | SAMN05773092 | blood | Dößelmann *et al.,* 2017 | GCF_002215345.1 | NZ_CP017293.1 |
| AR_0110 | 6402 | SAMN04014951 | missing | Conlan *et al.,* 2018 | GCF_003204335.1 | NZ_CP029745.1 |

Accession numbers available at the public database: CDC & FDA Antibiotic Resistance (AR) Isolate Bank. https://www.cdc.gov/drugresistance/resistance-bank/index.html
